# Human Brainstem and Cerebellum Atlas: Chemoarchitecture and Cytoarchitecture Paired to MRI

**DOI:** 10.1523/JNEUROSCI.0587-22.2022

**Published:** 2023-01-11

**Authors:** Lindsay J. Agostinelli, Scott C. Seaman, Clifford B. Saper, Dustin P. Fykstra, Marco M. Hefti, Timothy R. Koscik, Brian J. Dlouhy, Alexander G. Bassuk

**Affiliations:** ^1^Department of Neurology, University Pennsylvania, Philadelphia, Pennsylvania 19104; ^2^Stead Family Department of Pediatrics, Roy J. and Lucille A. Carver College of Medicine, University of Iowa Hospitals and Clinics, Iowa City, Iowa 52242; ^3^Department of Neurosurgery, University of Iowa, Iowa City, Iowa 52242; ^4^Department of Neurology, Beth Israel Deaconess Medical Center, Harvard Medical School, Boston, Massachusetts 02215; ^5^Department of Pathology, University of Iowa, Iowa City, Iowa 52242; ^6^Department of Psychiatry, University of Iowa, Iowa City, Iowa 52242; ^7^Department of Neurology, University of Iowa, Iowa City, Iowa 52242; ^8^Iowa Neuroscience Institute, University of Iowa, Iowa City, Iowa 52242

**Keywords:** acetylcholine, catecholamine, dopamine, melanin, norepinephrine, serotonin

## Abstract

Lesion localization is the basis for understanding neurologic disease, which is predicated on neuroanatomical knowledge carefully cataloged from histology and imaging atlases. However, it is often difficult to correlate clinical images of brainstem injury obtained by MRI scans with the details of human brainstem neuroanatomy represented in atlases, which are mostly based on cytoarchitecture using Nissl stain or a single histochemical stain, and usually do not include the cerebellum. Here, we report a high-resolution (200 μm) 7T MRI of a cadaveric male human brainstem and cerebellum paired with detailed, coregistered histology (at 2 μm single-cell resolution) of the immunohistochemically stained cholinergic, serotonergic, and catecholaminergic (dopaminergic, noradrenergic, and adrenergic) neurons, in relationship to each other and to the cerebellum. These immunohistochemical findings provide novel insights into the spatial relationships of brainstem cell types and nuclei, including subpopulations of melanin and TH^+^ neurons, and allows for more informed structural annotation of cell groups. Moreover, the coregistered MRI-paired histology helps validate imaging findings. This is useful for interpreting both scans and histology, and to understand the cell types affected by lesions. Our detailed chemoarchitecture and cytoarchitecture with corresponding high-resolution MRI builds on previous atlases of the human brainstem and cerebellum, and makes precise identification of brainstem and cerebellar cell groups involved in clinical lesions accessible for both laboratory scientists and clinicians alike.

**SIGNIFICANCE STATEMENT** Clinicians and neuroscientists frequently use cross-sectional anatomy of the human brainstem from MRI scans for both clinical and laboratory investigations, but they must rely on brain atlases to neuroanatomical structures. Such atlases generally lack both detail of brainstem chemical cell types, and the cerebellum, which provides an important spatial reference. Our current atlas maps the distribution of key brainstem cell types (cholinergic, serotonergic, and catecholaminergic neurons) in relationship to each other and the cerebellum, and pairs this histology with 7T MR images from the identical brain. This atlas allows correlation of the chemoarchitecture with corresponding MRI, and makes the identification of cell groups that are often discussed, but rarely identifiable on MRI scan, accessible to clinicians and clinical researchers.

## Introduction

Lesion localization is the basis for understanding neurologic disease, which is predicated on neuroanatomical knowledge carefully cataloged from histology and imaging. As functionally connected networks become further characterized, brain atlases have become essential reference resources for both clinical and scientific inquiry. Among human brainstem atlases, previous work was mostly based on cytoarchitecture (which limits the delineation of cell groups to Nissl-stained neuronal morphology and packing) or a single histochemical stain (often acetylcholinesterase) (Sabin, 1901; Paxinos, 1995; Buttner-Ennever and Horn, 2014). Therefore, a detailed map of neurons expressing key brainstem neurotransmitters has yet to be documented in a single human brainstem resource. Additionally, many human brainstem atlases do not include the cerebellum, neglecting the spatial relationship between the cerebellum and brainstem nuclei. This relationship is often important when considering infratentorial pathology such as tumors, and the consequences of neurosurgical intervention. Furthermore, many histologic atlases are difficult to correlate with the less detailed MRI used in clinical settings. While work delineating cell groups onto MRI cross-sections has been performed, these have largely been limited to drawing nuclei onto MRI scans informed by prior, separate histologic work (Soria et al., 2011). Only recently has 7 Tesla (7T) imaging, which provides ultra-high spatial resolution, been performed on cadaveric specimens (Augustinack and van der Kouwe, 2016; Edlow et al., 2019; Adil et al., 2021).

A goal of this current work was to map the distribution of key brainstem cell types (cholinergic, serotonergic, and catecholaminergic neurons) in relationship to each other and the cerebellum. Additionally, we aimed to pair this detailed histology with 7T MRI. Our detailed chemoarchitecture and cytoarchitecture with corresponding high-resolution MRI builds on previous atlases of the human brainstem, and makes precise identification of brainstem and cerebellar cell groups involved in clinical lesions accessible for both laboratory scientists and clinicians alike.

## Materials and Methods

### Brain tissue history

An adult brain was provided by the Iowa Neuropathology Department per research protocol. All donors or donor next of kin consented to use of tissue for research under the applicable sections of Iowa state law. This study was reviewed by the University of Iowa Institutional Review Board and determined not to constitute human subjects research under the Revised Common Rule (determination #201706772). We picked a case without known brainstem pathology, and compared the brainstem to others in our laboratory to pick a representative case. The brain was from a 68-year-old male patient with a past medical history of multiple myeloma, Type 2 diabetes mellitus, hypertension, peripheral vascular disease, and tobacco use; the cause of death was complications secondary to atherosclerotic cardiovascular disease. The brainstem and cerebellum were sharply separated from the diencephalon. There was a postmortem interval of 36 h. The brain was removed from the skull and the brainstem and cerebellum were preserved and fixed by cannulating the left vertebral artery (right vertebral was ligated at its most distal aspect) and basilar artery with 24 gauge angiography catheters and then arterially perfused with 10% formalin followed by submersion fixation in formalin for ∼14 d. The subsequent 7T MRI scan and serial section histology of the entire brainstem and cerebellum showed no evidence of vascular infarct or other pathology.

### 7T MRI imaging technique

#### Brain preparation for imaging

To minimize motion and wave artifacts of a liquid-based medium, the brainstem was embedded in 3% agar in an MRI compatible container. A lumbar drain catheter was inserted into the cerebellomedullary fissure oriented toward the cerebral aqueduct, and 1% agar was injected to fill the fourth ventricle to minimize air artifacts in the fourth ventricle, as the high-resolution susceptibility weighted imaging (SWI) sequence is highly sensitive to the presence of air. The container holding the tissue and agar was then refrigerated at 4C° overnight, before being equilibrated to ambient temperature the following morning before imaging.

#### Image acquisition

**Figure 1.**
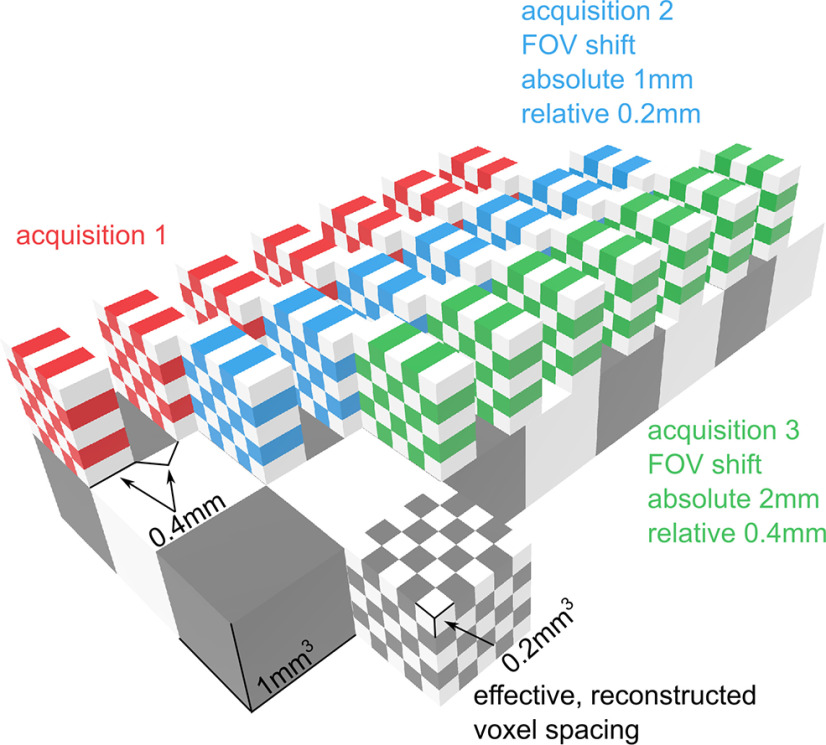
Schematic of the voxel-shift scanning procedure. Colored checkerboard blocks represent slices during separate image acquisitions: red represents acquisition 1; blue represents acquisition 2; green represents acquisition 3. Slices are thicker in the slicing plane (0.4 mm) than the acquisition plane (0.2 mm). Each acquisition is offset in the slice plane such that the slice planes differ by an amount similar to the acquisition plane resolution (0.2 mm). In this example, an offset of 1 mm between acquisitions results in a 0.2 mm slice offset between acquisitions given the 0.4 mm slice thickness and 0.4 mm slice spacing. Across the full set of acquisitions, the volume is measured with high in-plane resolution (gray checkerboard cube represents the effective reconstructed voxel spacing).

Images were acquired on a 7T GE Discovery MR950 scanner using a 32-channel head coil with a set of three anisotropic, susceptibility-weighted scans. Each scan was isotropic and had higher resolution in the acquisition plane (0.1758 × 0.1758 mm), but lower resolution out of this plane (0.4 mm); other scan parameters were as follows: TR = 66.3 ms, TE = 23.276 ms, flip angle = 15 degrees, FOV = 1024 × 1024, number of slices = 258, voxel resolution = 0.1758 × 0.1758 mm, slice thickness = 0.4 mm, slice spacing = 0.4 mm. In between each acquisition, the FOV was shifted in the plane perpendicular to the plane of acquisition by 1 mm. Since the slice thickness and slice gap total 0.8 mm, this 1 mm shift means that slices between acquisitions are staggered by 0.2 mm, such that across the three scans the physical space is fully sampled at ∼0.2 mm^3^ and can be reconstructed at this resolution (Fig. 1).

#### Image reconstruction

After acquisition, images were processed using image processing procedures available in Advanced Normalization Tools (Avants et al., 2011). First, images were preprocessed independently to remove noise, using an adaptive nonlocal procedure and a Rican noise model and nonuniform intensity because of regional inhomogeneity in the magnetic field, using the N4 procedure (Manjon et al., 2010; Tustison et al., 2010). After images were cleaned in their native, anisotropic space, they were each resampled to exhibit isotropic resolution at the smallest voxel dimension (∼0.2 mm^3^), using a third-order B-spline interpolation. Images were then coregistered using a 4-stage registration in the following order: (1) translation, (2) rigid with 6 degrees of freedom, (3) low-resolution affine with 12 degrees of freedom, and (4) high-resolution affine. Coregistration was initialized by generating an average of all resampled images and using this as the fixed target for registration. For each subsequent coregistration step, the output coregistered images of the prior step were averaged to generate a new fixed target for registration; this iteratively improves the average for each step. The averages of the final coregistration step constitute the isotropically reconstructed image. If acquisitions are perfect, translation or rigid registration would be all that was necessary; however, affine registration adds in correction for differences in image distortion that may be present because of the different FOV.

### Histology

After MRI imaging, the tissue was then sectioned axially in the agar into 4 mm tissue blocks. We then selected the first 10 blocks for further dissection, since they contained the majority of the brainstem. These blocks were incubated in a 20% sucrose solution for ∼48 h or until the blocks sunk. Then, using a freezing microtome, we sectioned the tissue into 40 μm slices, and we collected at least four sections from each block. One section from each block was mounted on either a 2” × 3” or 5” × 7” gelatinized slide for Nissl staining. The rest of the tissue was placed in a cryoprotection solution and stored at −20°C for later immunohistochemistry staining.

To fully assess each block, four adjacent sections of each block were stained to visualize Nissl, catecholaminergic, cholinergic, or serotonergic neurons. To visualize the cytoarchitecture, we used the first section cut from each block to perform a Nissl stain with thionine. These slide-mounted sections were submerged in water for 1 min followed by 3 min in thionine. Slides were then rinsed in water until the water ran clear, and then we dehydrated sections in a graded ethanol series for 3 min each, and cleared in xylenes before coverslipping with a toluene-based mounting media (Cytoseal; Thermo Fisher Scientific).

We then took the next three adjacent sections from each block and prepared them for immunohistochemistry. First, we performed antigen retrieval by boiling the sections in a solution of 0.1 m sodium citrate for 10 min and then rinsing in PBS. We then took the second, third, and fourth section from each block and incubated it overnight in either rabbit anti-tyrosine hydroxylase (TH) (1:1,000; Millipore; AB152), goat anti-choline acetyltransferase (ChAT) (1:100; Millipore, AB144P), or mouse anti- tryptophan hydroxylase (TpOH) (1:1,000; Millipore, MAB5278) to label catecholaminergic, cholinergic, and serotonergic cells, respectively. We then placed sections in biotinylated donkey anti-goat secondary antiserum (1:500, Jackson ImmunoResearch, #705-065-147), biotinylated donkey anti-goat IgG secondary antiserum (1:500, Jackson ImmunoResearch, #705-065-147), or biotinylated donkey anti-mouse (1:500, Jackson ImmunoResearch, #715-066-150) followed by 1 h in avidin-biotin complex (Vectastain ABC Elite Kit, Vector Laboratories). We made DAB solution in TBS containing 0.024% hydrogen peroxide, and modified the DAB to produce a black stain by adding 0.2% ammonium nickel (II) sulfate (Sigma) to the DAB solution. After immunolabeling, we mounted and dried sections on 5” × 7” or 2” × 3” slides. We dehydrated sections in graded ethanols for 3 min each and cleared in xylenes before coverslipping with a toluene-based mounting media (Cytoseal, Thermo Fisher Scientific).

### Microscope imaging and mapping

We acquired whole-slide images using a slide-scanning microscope (Olympus VS120, Shinjuku) using 10× and 20× objectives and extended focal imaging. Extended focal imaging makes a *z*-series of images through the tissue and combines them into one in-focus image. We reviewed images in Olympus' OlyVIA software. We placed images of sections from OlyVia into Adobe Illustrator; and for each block, we layered the images from adjacent tissue sections containing the immunohistochemical stains on top of the corresponding Nissl section. We then graphically marked each DAB-stained neuron with different color dots (orange represents ChAT stain; pink represents TpOH stain; lime green represents TH stain; dark green represents melanin-pigmented), to map the spatial relationship of different cell types. These dots are larger than the actual cells to allow for visibility in the zoomed-out images. Additionally, these dots usually represent a single neuron; but in a few cases when neurons were too densely packed, a single dot might overlay 2 or 3 neurons. We used dashed lines to indicate rough outlines of nuclei, and solid lines for the more well-defined white matter of axons/fiber tracts (that was well defined in the Nissl or immunohistochemical stains). To highlight cranial nerves, we traced ChAT^+^ axons with solid, transparent gray lines. In general, we labeled nuclei with capital letters and fiber tracts with lowercase letters. Cranial nerve nuclei are labeled with roman numerals, and their corresponding nerves are labeled with the roman numeral + n (e.g., VII for facial motor nucleus and VIIn for facial nerve or cranial nerve 7). The Appendix contains all of the abbreviations for structures mentioned in the figures and the text.

### Imaging histologic coregistration

After final MRI reconstruction using Advanced Normalization Tools, the original block slabs were coregistered to the MRI using manual registration. Then the MRI was coregistered to each individual axial histologic section from each of the 10 blocks in ItK-SNAP (Yushkevich et al., 2006) (www.itksnap.org, version 3.8.0). These axial histology levels are demonstrated on a sagittal image to help show the rostrocaudal distribution (see Fig. 5). We then identified the rostral tip of the inferior olive (IO) as point zero, and then calculated the distance of each histologic section from the IO zero point. This MRI image of each histologic section was then exported for imaging segmentation and structure labeling.

## Results

Overall, we were able to robustly label cholinergic, serotonergic, and catecholaminergic neurons, axons, and terminal fields throughout the human brainstem (Figs. 2–4). For each level, we mapped all of these cell types and tracts onto one Nissl-stained section to visualize their spatial relationships (Figs. 6–25).

**Figure 2.**
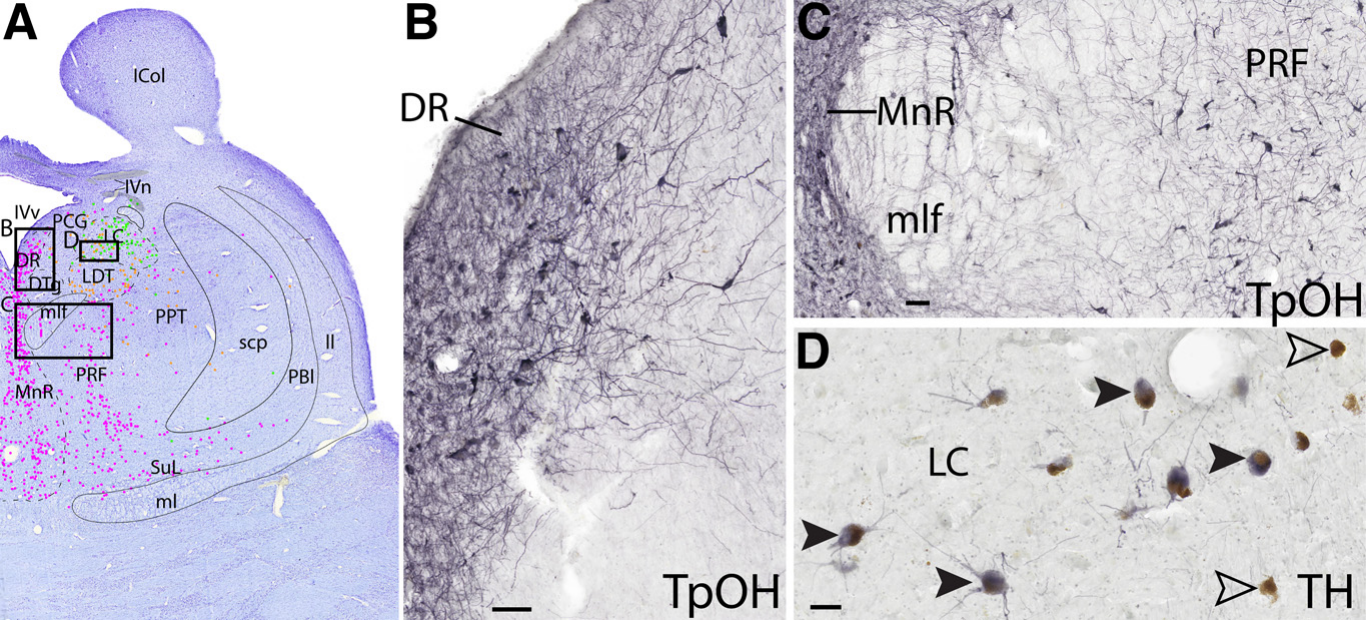
Example of staining serotonergic and catecholaminergic neurons. ***A***, Level C Nissl stain with dots representing each cell type (further shown in Figs. 7, 8). Black boxes in ***A*** represent panels ***B*** and ***C***. ***B***, Serotonergic neurons (TpOH) in the DR are robustly labeled. Scale bar, 100 μm. ***C***, Serotonergic neurons are smaller in the MnR and are larger laterally in the PRF. ***D***, Most of the brown pigmented adrenergic neurons of the LC also stain black with TH (solid black arrows), but some melanin pigmented neurons do not stain with TH (hollow arrows). Scale bars, 50 μm.

Briefly, TpOH robustly and consistently labels serotonergic neurons, including fibers (Fig. 2*B*,*C*). This allows for easy visualization of the raphe nuclei and surrounding white matter tracts.

The catecholaminergic neurons (described further in Catecholaminergic groups) are marked by TH stain, melanin pigment, or both (Figs. 2*D*, 4*C*). We marked the cells lime green if they were TH^+^, regardless of whether they were also pigmented with melanin, but we labeled them darker green if they were pigmented and TH^–^. Immunofluorescence is typically used to view colocalization; but when looking at this tissue under a microscope, this was easily visualized. The melanin pigment is brown, granular, and mostly concentrated in only a portion of the neurons' cytoplasm; this contrasts to the more translucent greyish-black TH stain in the cytoplasm that surrounds the melanin (filling up the entire cytoplasm) in those neurons in which the two markers colocalize (Fig. 2*D*). In general, we found that the TH^+^ neurons that are nonpigmented stain more darkly for TH than the TH^+^ cells that contain pigmented melanin, and the most darkly pigmented melanin neurons appear to be TH^–^ (or their TH levels are too low to detect). The best example of the darkest melanin-pigmented neurons is in the substantia nigra (SN), and most of these pigmented neurons are TH^–^ (Figs. 6, 7). The nucleus of the solitary tract (NTS)/C2 is an example of robustly stained TH^+^ neurons that lack melanin, and ventrolaterally, there are two large melanin-pigmented neurons without TH staining (Fig. 4*C*).

**Figure 3.**
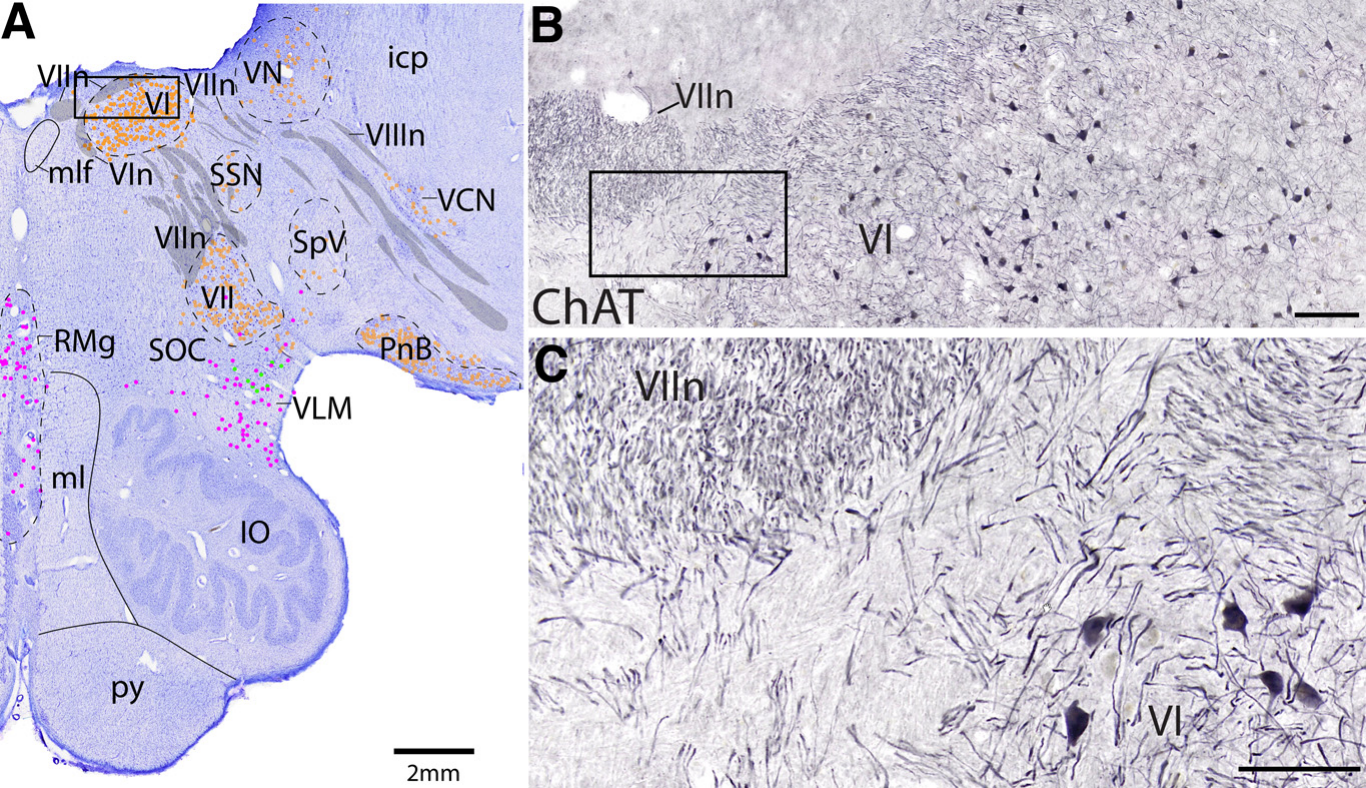
Example of ChAT stain used to identify cranial nerves and nuclei. ***A***, Level G Nissl stain with dots representing each cell type (further shown in Figs. 18, 19). Black box represents panel ***B***. ***B***, ***C***, ChAT stain revealing cholinergic fibers of cranial nerve 7, facial nerve, wrapping around motor neurons and axons of cranial nerve 6, abducens nerve. Scale bars: ***B***, 200 μm; ***C***, 100 mm.

**Figure 4.**
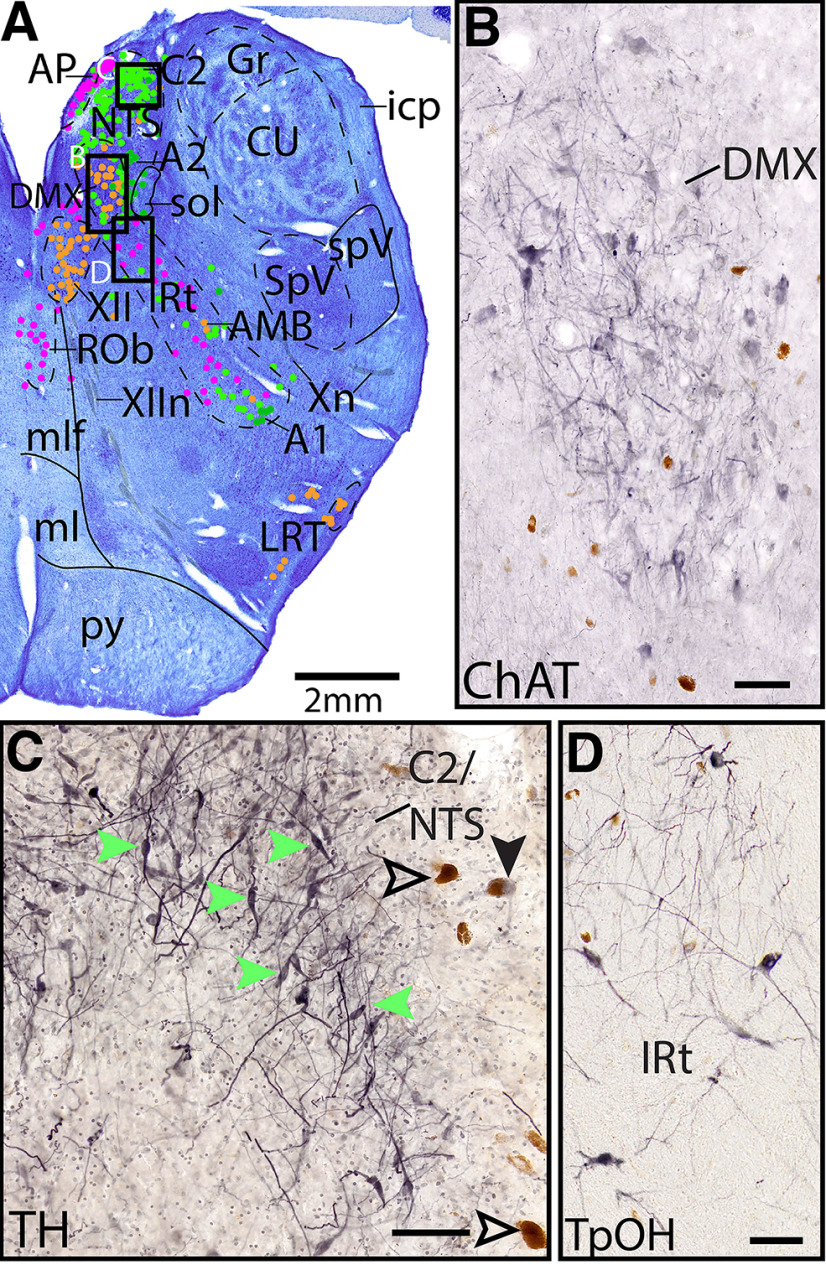
Individual ICH stains were used to map different cell types. ***A***, Example section (Level I, further shown in Figs. 22, 23) Nissl section with dots used to represent each cell type (orange represents ChAT; lime green represents TH; dark green represents melanin; pink represents TpOH). Boxes represent location of ***B–D***. ***B–D***, Adjacent section stained blackish purple with DAB for ChAT, TH, or TpOH, respectively. Brown cells are endogenous pigmented melanin. ***D***, Most of the TH^+^ NTS neurons do not contain melanin (examples marked with green arrows), but one cell contains both melanin and TH (solid black arrow), and some cells with melanin are TH^–^ (hollow arrows). Scale bars, 100 μm.

Robustly labeled ChAT^+^ cholinergic neurons are found in motor nuclei, preganglionic parasympathetic nuclei (preganglionic Edinger–Westphal [EW] nucleus, salivatory nuclei), the pedunculopontine tegmentum (PPT), the laterodorsal tegmentum (LDT), the lateral parabrachial nucleus, and the reticular formation. The largest and most numerous cholinergic cells were located within the motor nuclei of cranial nuclei, particularly those of cranial nuclei 3, 5, 6, 7, 10, and 12 (Figs. 3*B*,*C*, 4*B*). Robustly labeled ChAT^+^ fibers were useful in identifying cranial nerves (e.g., the facial nerve wrapping around the abducens nucleus) (Fig. 3).

The location of each axial brainstem level is mapped in Figure 5. The levels are ∼4 mm apart. We paired each histologic level with the corresponding level on MRI (Figs. 6–25). These 7T MRI sequences have 200 μm resolution. The MRI SWI images represent axonal white matter as dark gray (e.g., superior cerebellar peduncle (scp) in Levels D-F or medullary pyramids (py) on Level F) and gray matter/nuclei as lighter gray (IO, Levels G–H).

**Figure 5.**
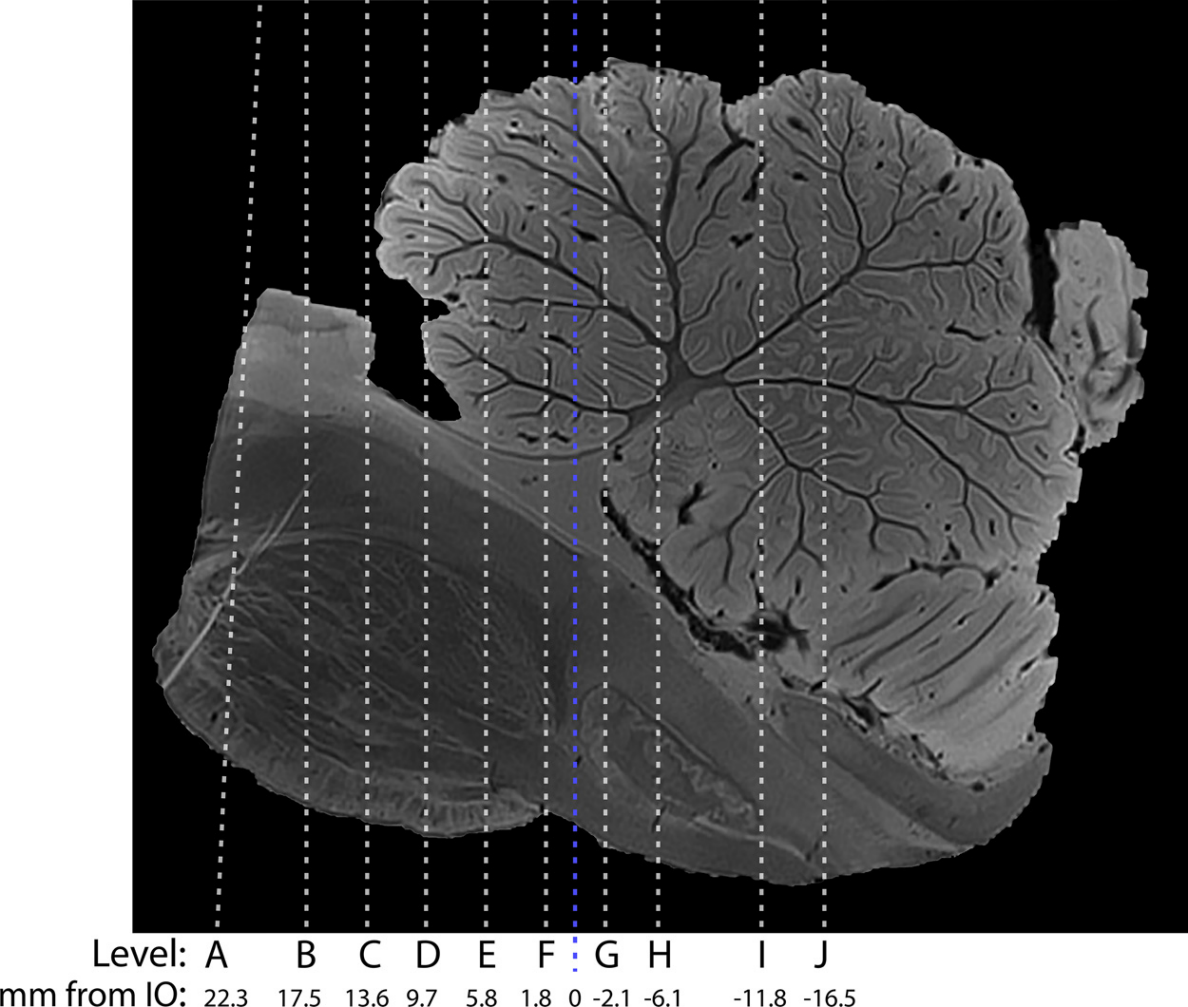
Sagittal MRI image demonstrating the location of each histologic level, and their distances from the anterior inferior olive (blue line). Level A is referenced in Figures 6 and 7. Level B is referenced in Figures 8 and 9. Level C is referenced in Figures 2, 10, and 11. Level D is referenced in Figures 12 and 13. Level E is referenced in Figures 14 and 15. Level F is referenced in Figures 16 and 17. Level G is referenced in Figures 3, 18, and 19. Level H is referenced in Figures 20 and 21. Level I is referenced in Figures 4, 22, and 23. Level J is referenced in Figures 24 and 25.

**Figure 6.**
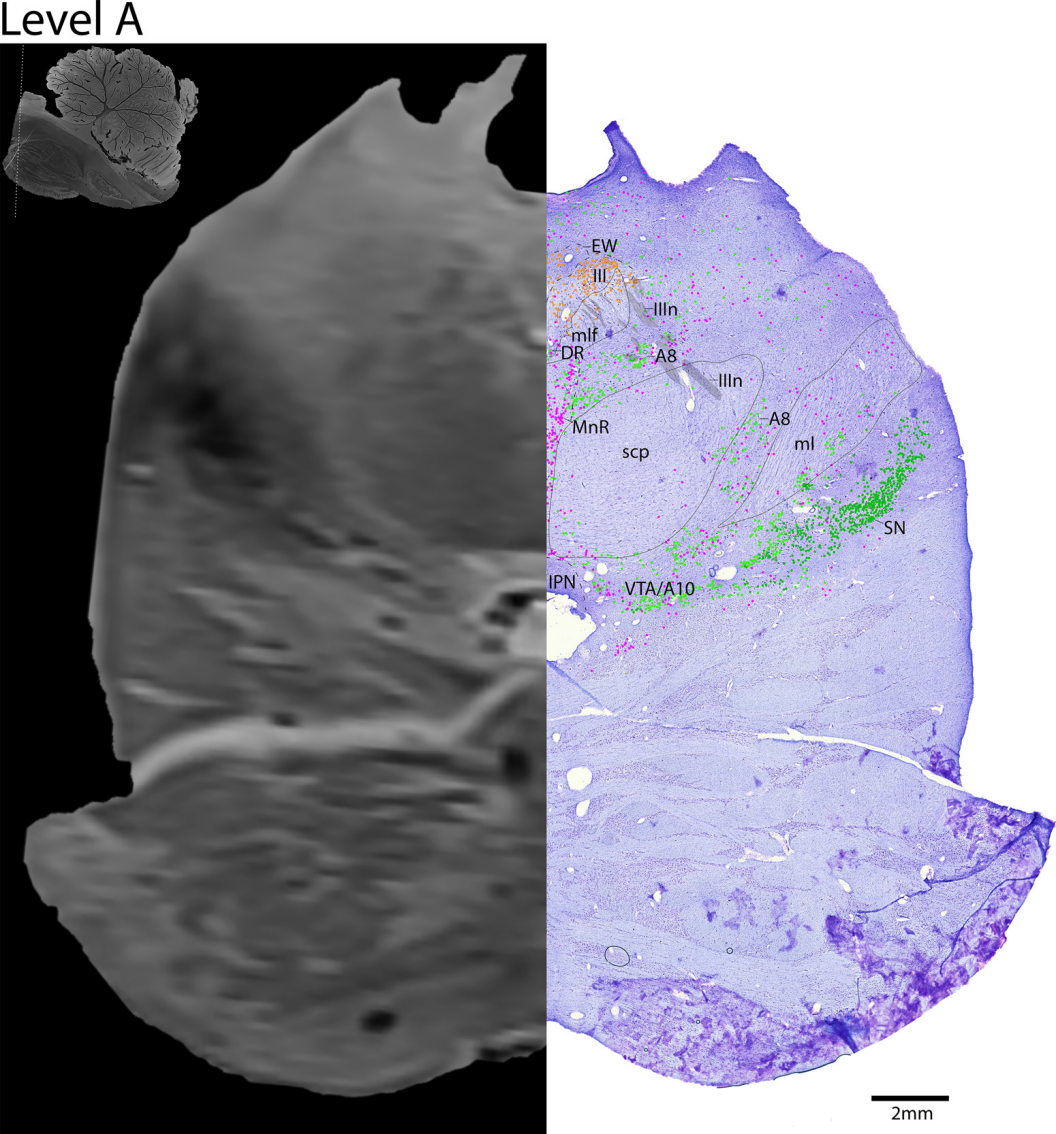
The upper midbrain. Level A is comprised of MRI image on left and Nissl stain on right. Dots represent different cell types (orange represents ChAT; pink represents TpOH; bright green represents TH; dark green represents melanin-pigmented). Dashed line through sagittal section indicates the rostrocaudal level.

In terms of mapping the different cell types, we hesitate to draw circles around cell groups since many do not have obvious, crisp borders, such as the diffuse serotonergic neurons across the lateral pons. Additionally, some cell groups overlap more than we expected, such as the cholinergic neurons of the LDT intermingling with the catecholaminergic neurons of the locus coeruleus (LC) (Figs. 10, 11). Therefore, we limited the dashed line drawings to more obvious nuclei, and used dots representing neurons to demarcate the less defined regions. Furthermore, the boundaries between some nuclei identified by cytoarchitecture are less obvious given the lack of distinct chemogenic markers, retrograde tracing, or functional data in postmortem human brains, such as between the superior and inferior colliculi (Figs. 8, 9). In this case, we identified the superior colliculus by the clear laminations (as in other species), with smaller, densely packed cells in the outer layer, medium sized cells in the middle layer, and larger cells in the deeper layer, while the inferior colliculus was identified by the central nucleus surrounded by a fibrous lamina. The exact boundary between these nuclei is unclear with cytology alone.

We subdivided the cerebellar cortex into the cerebellar vermis in the center, and cerebellar hemisphere laterally using cytoarchitecture (Figs. 10, 12, 14, 16, 18, 20, 22, 24), and further identify the flocculus on the ventral surface (Fig. 18). Within the vermis, we subdivided the parallel layers to show the outer, moderately stained molecular layer, the narrow middle layer containing Purkinje cells, and the inner, densely packed and deeply stained granular cell layer (Fig. 16). We delineated the deep cerebellar nuclei based on cytoarchitecture. From medial to lateral, they include the following: fastigial, globose, emboliform, and dentate nuclei (Figs. 20, 22, 24).

Here we will briefly review the main structures on each Levels A-J (Figs. 6–25), but the Discussion provides details about how each delineation was determined and comparison with prior literature. The caudal midbrain is located in the dorsal half of Levels A and B, and the ventral half contains the rostral portion of the basis pontis and corticospinal tract. Levels C-G contain the bulk of the pons and the rostral cerebellum. Levels H-J contain the majority of the medulla and the widest portion of the cerebellum.

**Figure 7.**
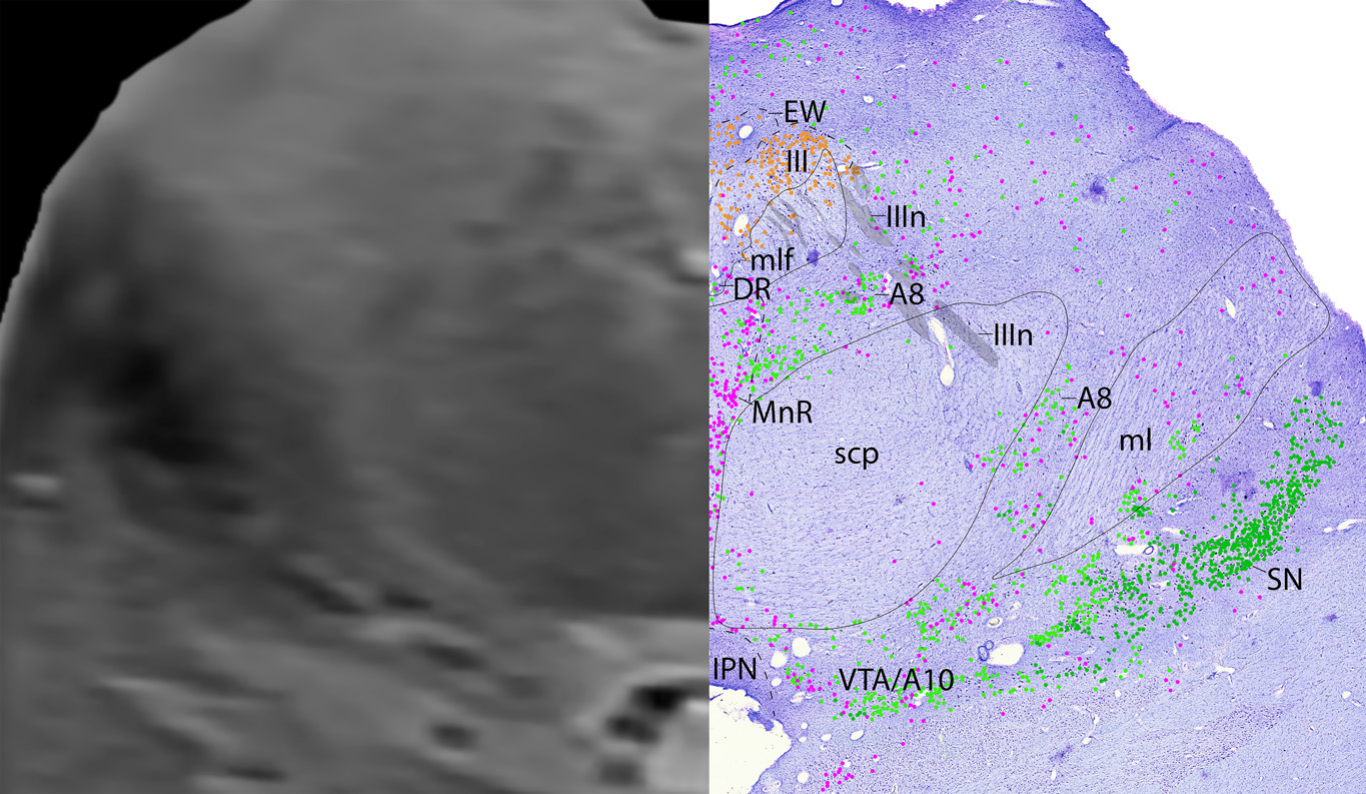
The upper midbrain, a zoomed-in view. This is a zoomed-in view of the brainstem of Level A from Figure 6.

*Level A*: The upper midbrain (Figs. 6, 7) contains the catecholaminergic (dopaminergic) SN laterally, and VTA medially. Dorsal to these structures are adrenergic A8 cells that surround the superior cerebellar peduncles (scp) more laterally. In the midline lies the rostral portion of the serotonergic dorsal raphe (DR) neurons. Dorsal to the DR is the third cranial nucleus (III). The oculomotor nucleus (III) is identified by large cholinergic somata in the midbrain with ChAT^+^ fibers exiting ventrally (IIIn), as they eventually exit out of the base of the midbrain. The parasympathetic neurons of III, which form the preganglionic EW nucleus, are identified by the smaller ChAT^+^ neurons situated just dorsally to the more tightly clustered IIIB.

**Figure 8.**
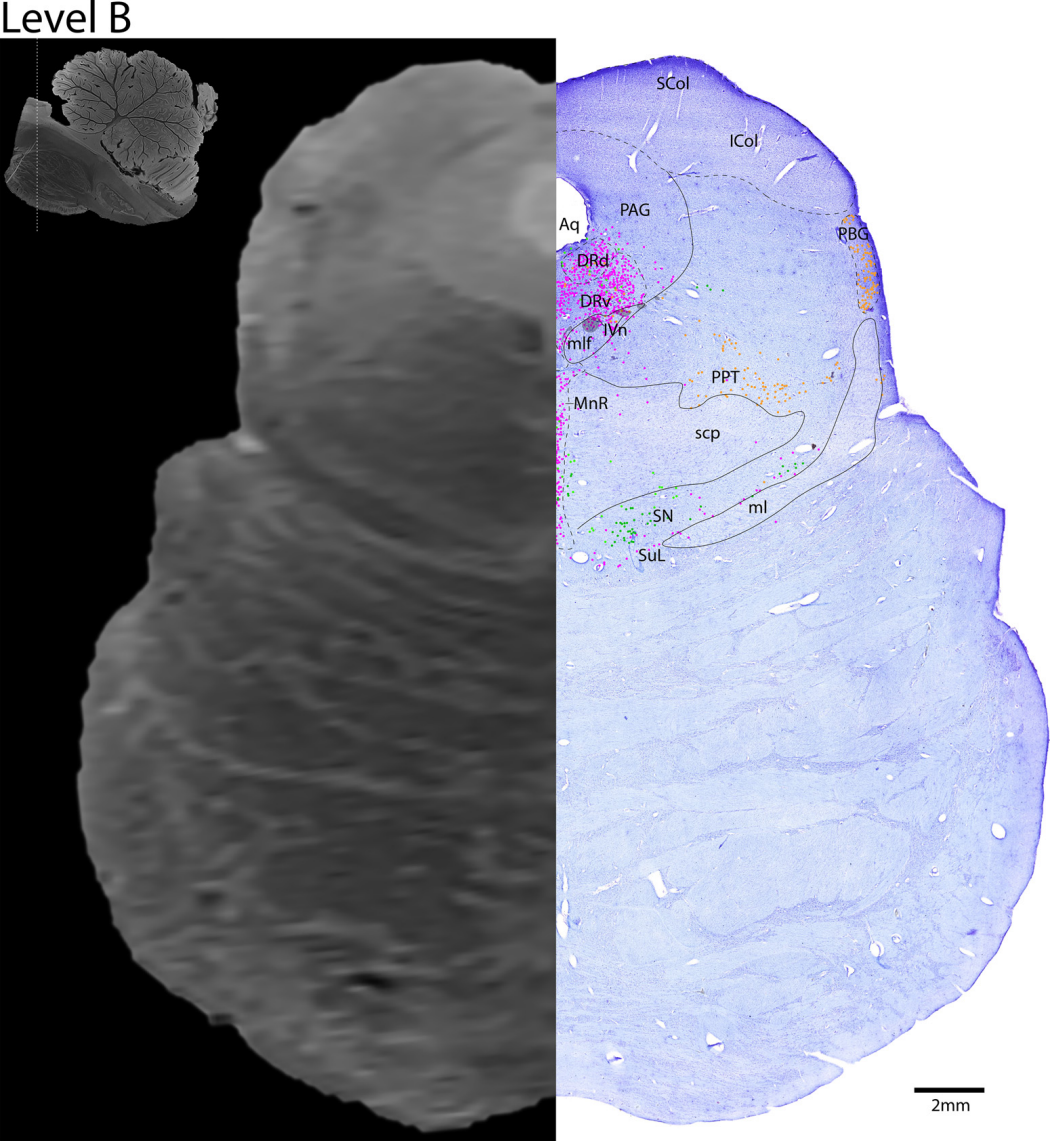
The caudal midbrain. Level B is comprised of MRI image on left and Nissl stain on right. Dots represent different cell types (orange represents ChAT; pink represents TpOH; bright green represents TH; dark green represents melanin-pigmented). Dashed line through sagittal section indicates the rostrocaudal level.

**Figure 9.**
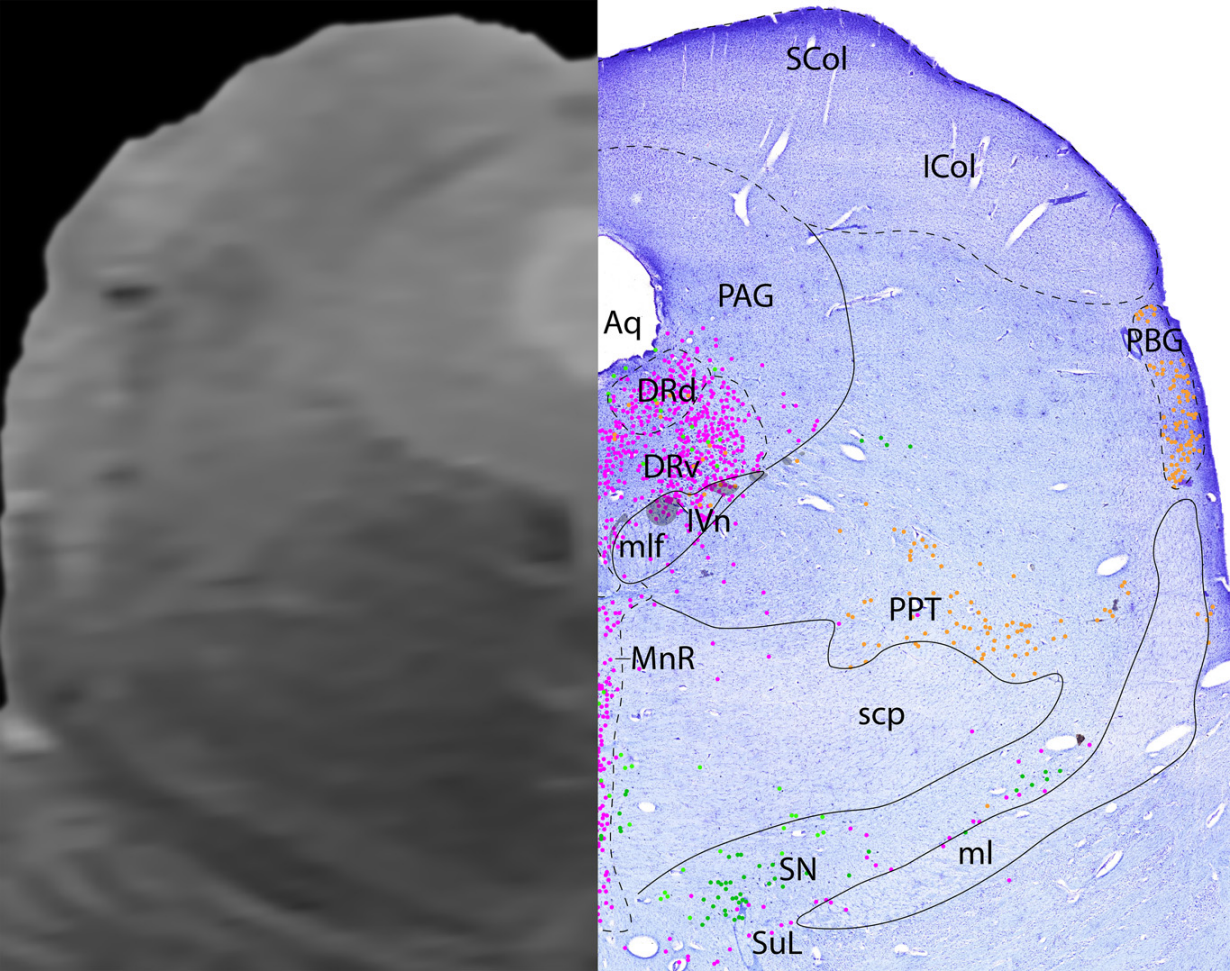
The caudal midbrain, a zoomed-in view. This is a zoomed-in view of the brainstem of Level B from Figure 8.

*Level B*: The caudal midbrain level (Figs. 8, 9) contains the superior colliculus dorsomedially, and inferior colliculus laterally. Ventral to the to the colliculi, the periaqueductal gray surrounds the aqueduct. Along the midline is the largest portion of the serotonergic DR and the more ventral median raphe (MnR). Ventral to the DR are the cholinergic fibers of cranial nerve 4, the trochlear nerve (IVn). Additionally, this level contains the cholinergic PPT, and the cholinergic parabigeminal nucleus laterally.

**Figure 10.**
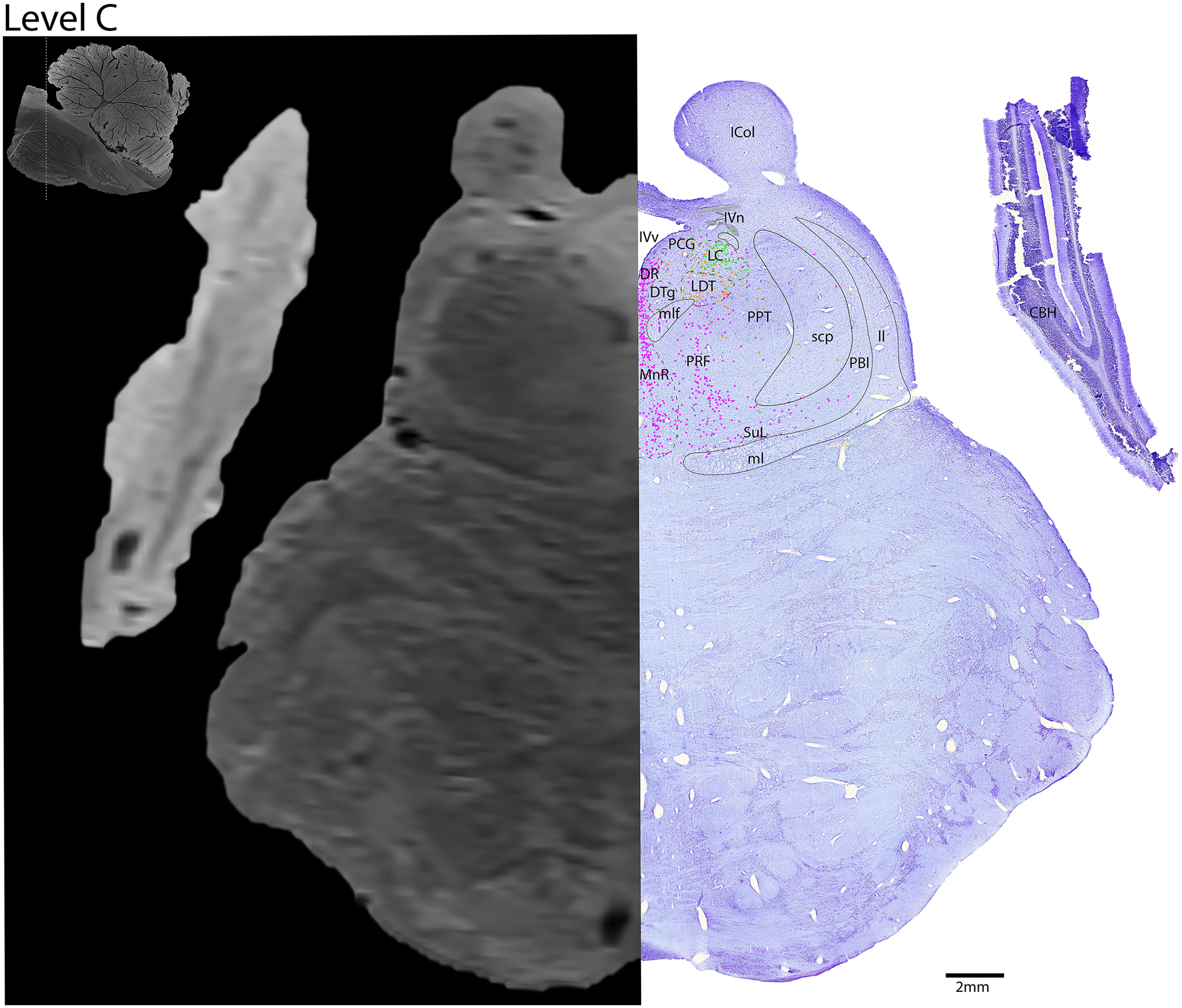
The midbrain-pontine junction. Level C is comprised of MRI image on left and Nissl stain on right. Dots represent different cell types (orange represents ChAT; pink represents TpOH; bright green represents TH; dark green represents melanin-pigmented). Dashed line through sagittal section indicates the rostrocaudal level.

**Figure 11.**
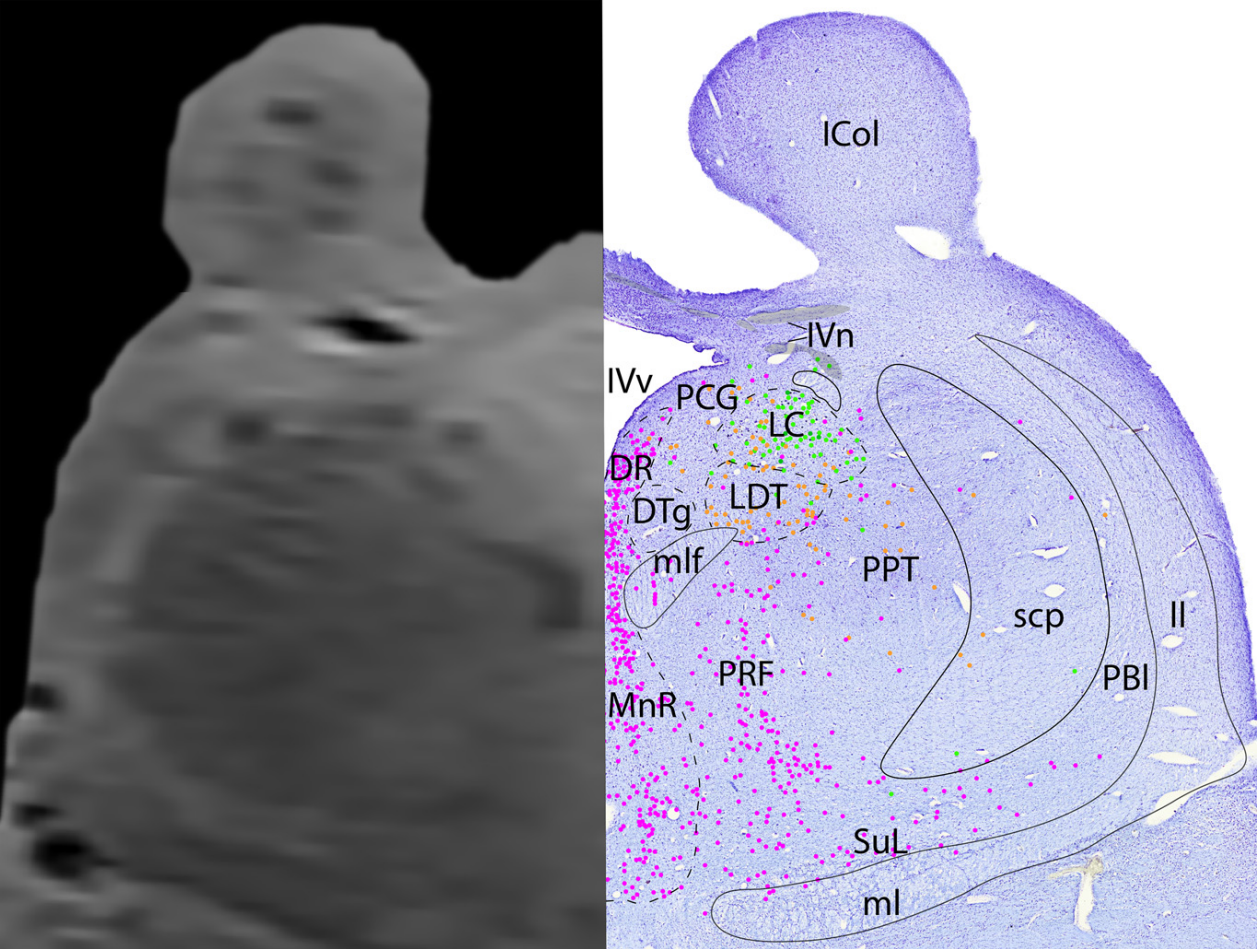
The midbrain-pontine junction, a zoomed-in view. This is a zoomed-in view of the brainstem of Level C from Figure 10.

*Level C*: The midbrain-pontine junction level (Figs. 10, 11) contains the rostral portion of the adrenergic LC, which intermingles with the cholinergic neurons of the LDT nucleus ventrally. Lateral to the LDT is the caudal portion of the cholinergic PPT. Further dorsally, the cholinergic fibers of IVn can be seen decussating over the fourth ventricle (IVv), ventral to the inferior colliculus. In the midline are a narrower portion of the serotonergic DR, and a larger portion of the MnR ventrally. Lateral to the MnR is the more diffuse pontine reticular formation (PRF) and supralemniscal/B9 nucleus (SuL).

**Figure 12.**
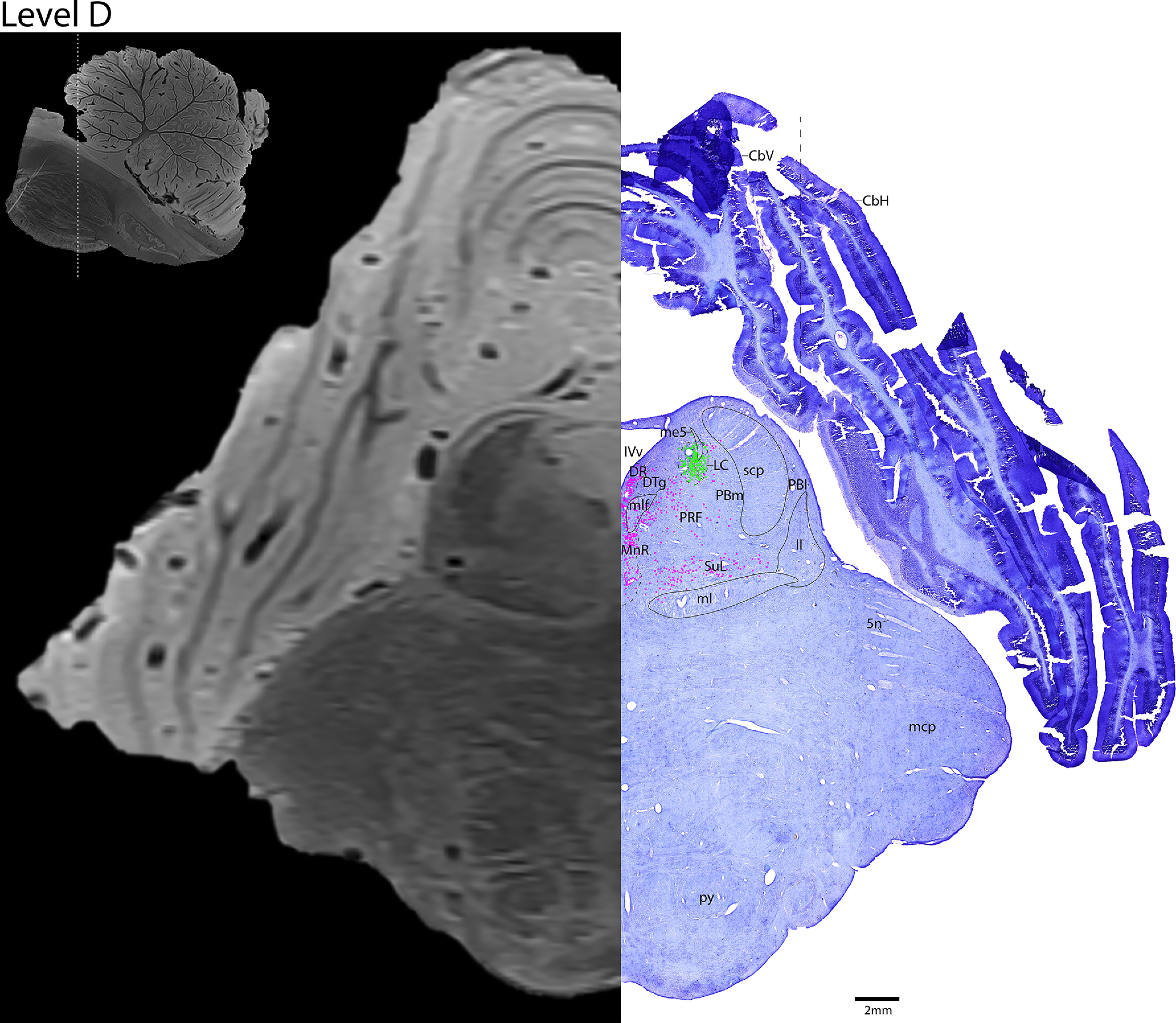
The rostral pons. Level D is comprised of MRI image on left and Nissl stain on right. Dots represent different cell types (orange represents ChAT; pink represents TpOH; bright green represents TH; dark green represents melanin-pigmented). Dashed line through sagittal section indicates the rostrocaudal level.

**Figure 13.**
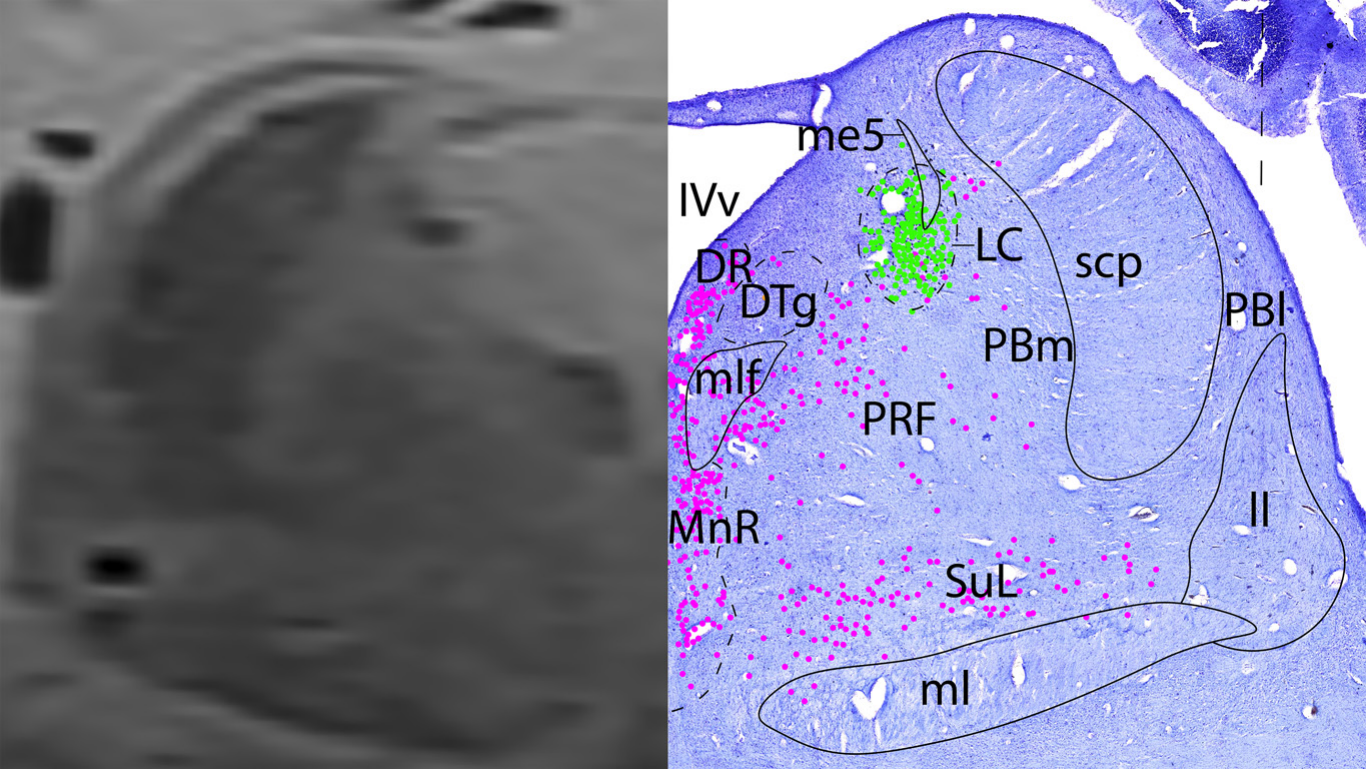
The rostral pons, a zoomed-in view. This is a zoomed-in view of the brainstem of Level D from Figure 12.

*Level D*: The rostral pontine level (Figs. 12, 13) contains the middle of the adrenergic LC, which contains tightly clustered and robustly stained TH^+^ neurons. Along the midline is the caudal serotonergic DR and MnR. Lateral to the MnR is the more diffuse serotonergic PRF and SuL, which run along the medial lemniscus (ml). The rostral cerebellum is present at this level.

**Figure 14.**
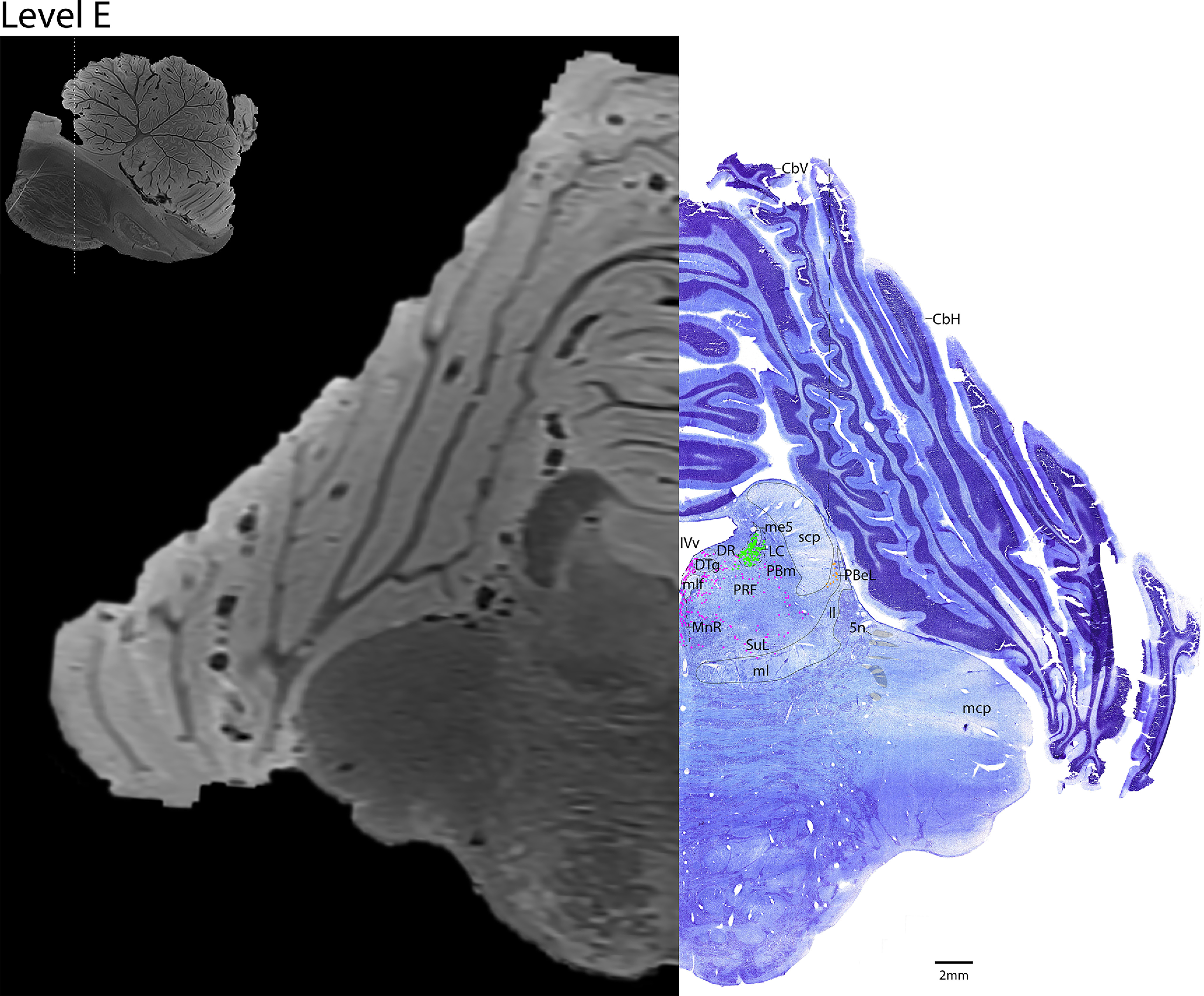
The mid-pons. Level E is comprised of MRI image on left and Nissl stain on right. Dots represent different cell types (orange represents ChAT; pink represents TpOH; bright green represents TH; dark green represents melanin-pigmented). Dashed line through sagittal section indicates the rostrocaudal level.

**Figure 15.**
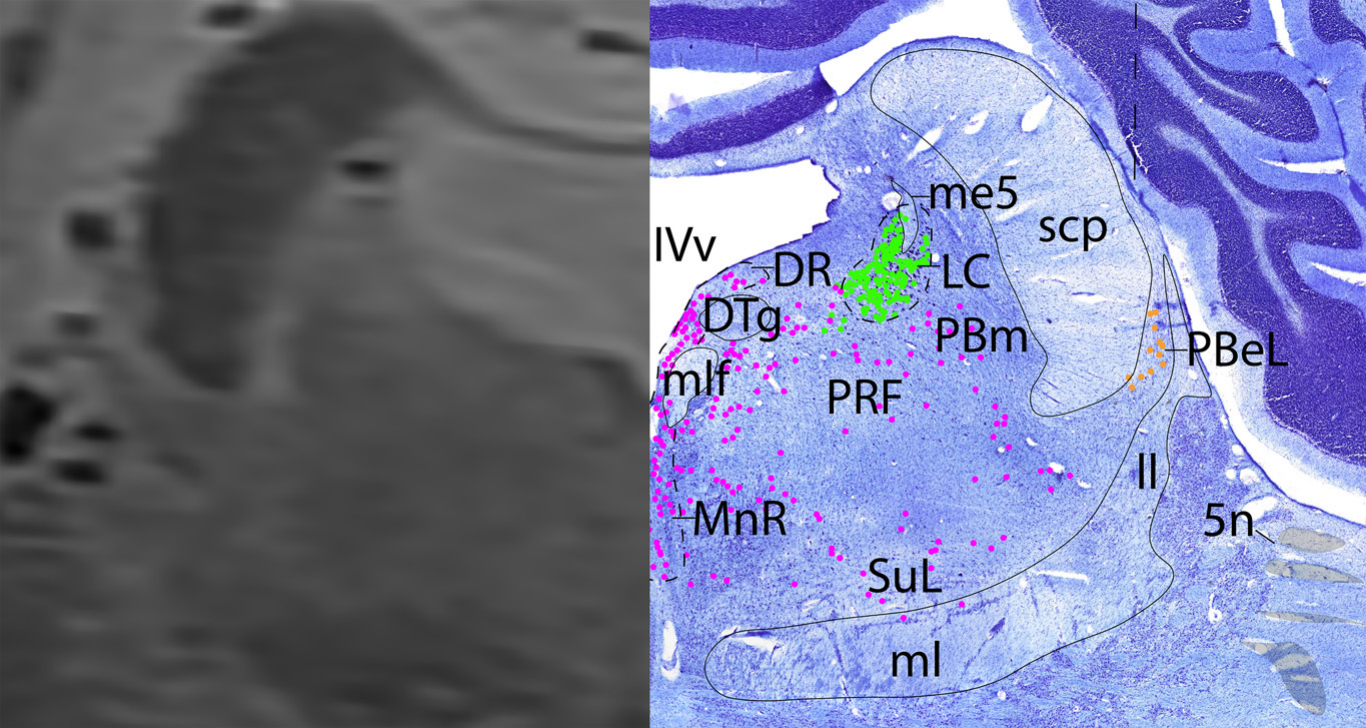
The mid-pons, a zoomed-in view. This is a zoomed-in view of the brainstem of Level E from Figure 14.

*Level E*: At the mid-pontine level (Figs. 14, 15) contains the densely clustered adrenergic neurons of the LC. The caudal portions of the serotonergic groups are present (DR and MnR midline, with the more diffuse PRF and SuL laterally). The medial parabrachial nucleus is medial to the crescentic superior cerebellar peduncle (scp). Lateral to the scp is the external lateral parabrachial nucleus, which contains a few cholinergic neurons. Additionally, the rostral portion of the cholinergic trigeminal nerve/cranial nerve 5 (Vn) can be seen traveling laterally through the middle cerebellar peduncle (mcp). There cerebellar vermis and hemispheres are present.

**Figure 16.**
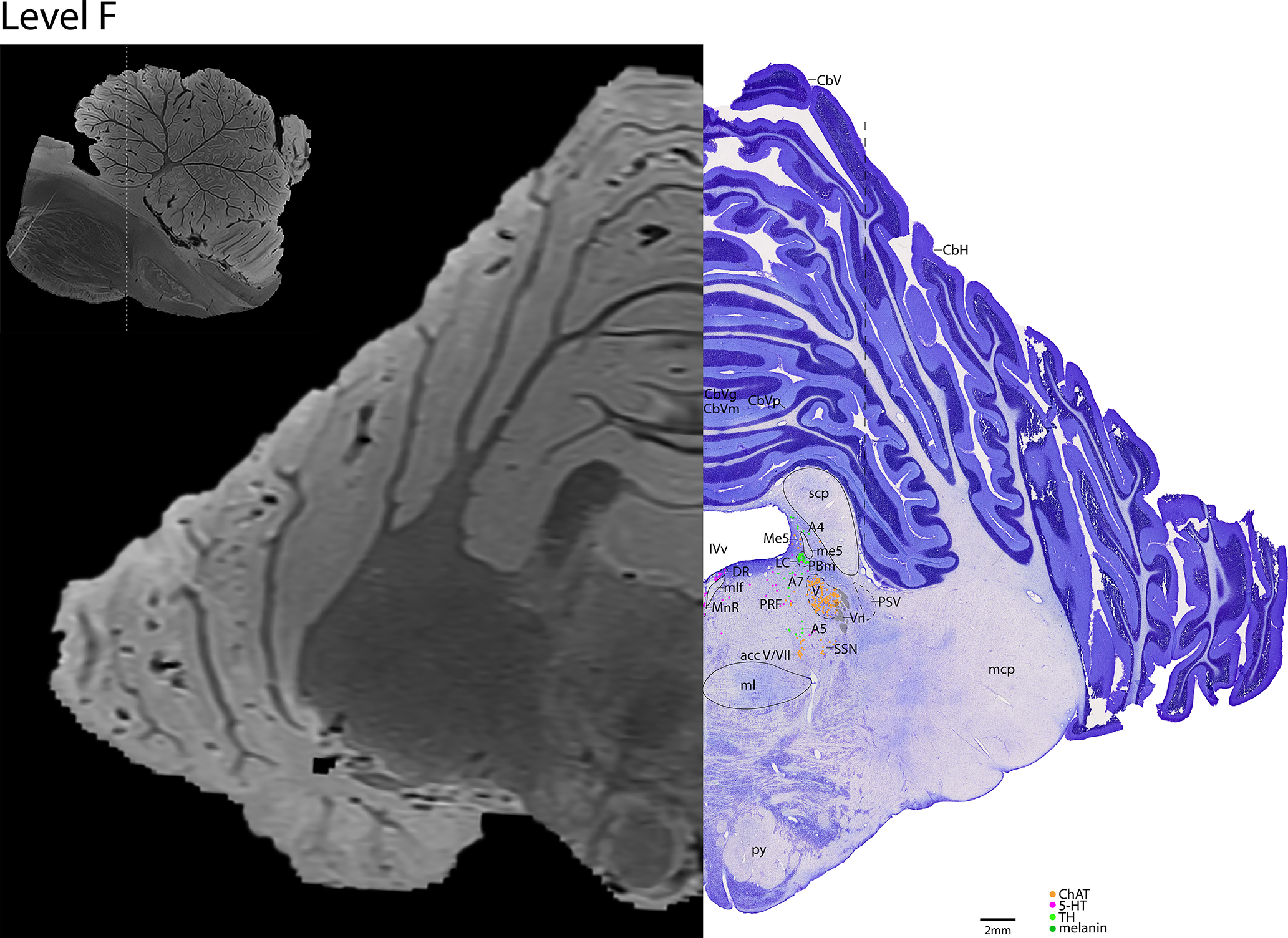
The caudal pons. Level F is comprised of MRI image on left and Nissl stain on right. Dots represent different cell types (orange represents ChAT; pink represents TpOH; bright green represents TH; dark green represents melanin-pigmented). Dashed line through sagittal section indicates the rostrocaudal level.

**Figure 17.**
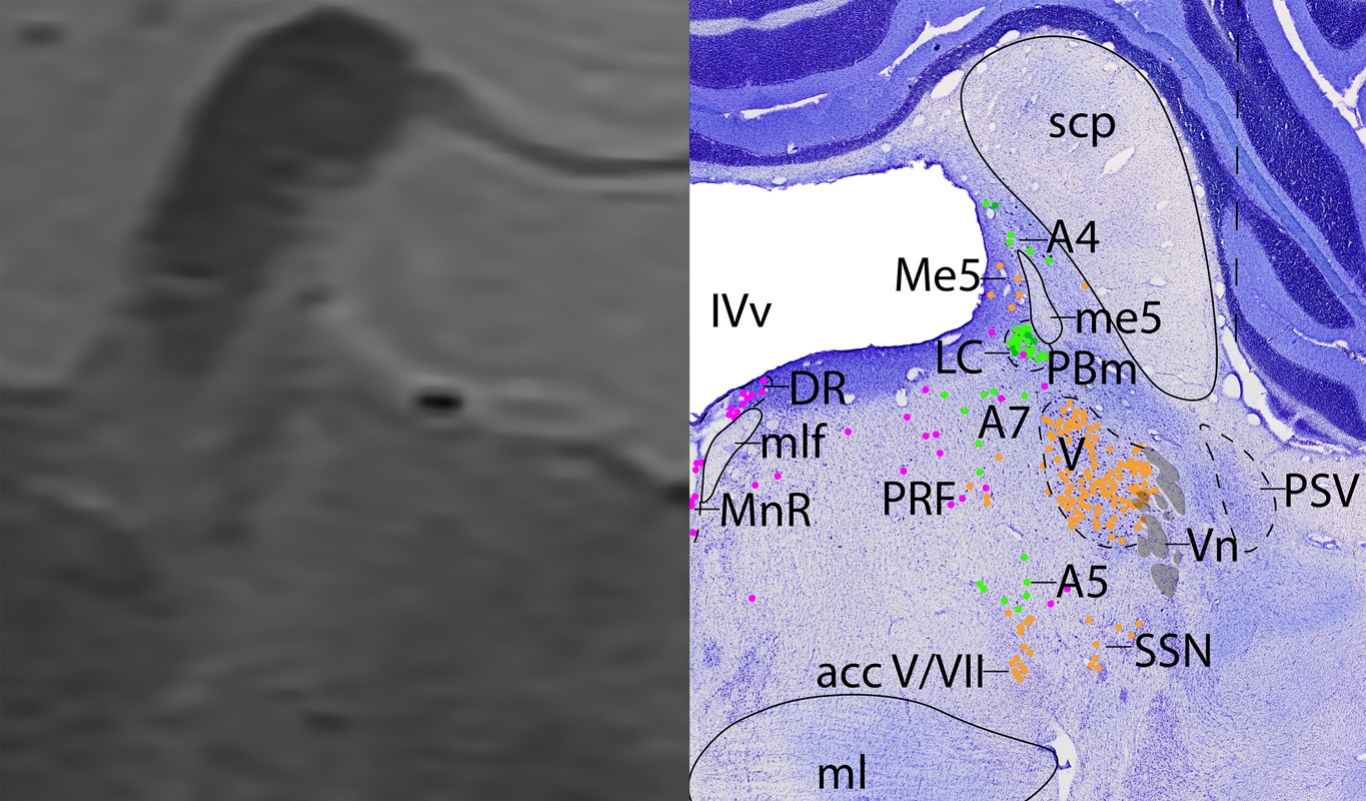
The caudal pons, a zoomed-in view. This is a zoomed-in view of the brainstem of Level F from Figure 16.

*Level F*: The caudal pontine level (Figs. 16, 17) is marked by the large cholinergic motor neurons of the trigeminal motor nucleus (V), and the cholinergic axons of cranial nerve 5 can be seen exiting the nucleus laterally (Vn). The principle sensory trigeminal nucleus is seen lateral to V. Ventral to V are the cholinergic accessory trigeminal and accessory facial neurons (acc V/VII), and the cholinergic superior salivatory nucleus (SSN). Dorsal to V are the caudal portion of the adrenergic LC and the medial parabrachial nucleus, while immediately ventral to V are the adrenergic A5 neurons. At this level, there are only a few serotonergic neurons of the DR, MnR, and PRF. At this level and one caudal, the cerebellum is attached to the brainstem via the middle cerebellar peduncle (mcp).

**Figure 18.**
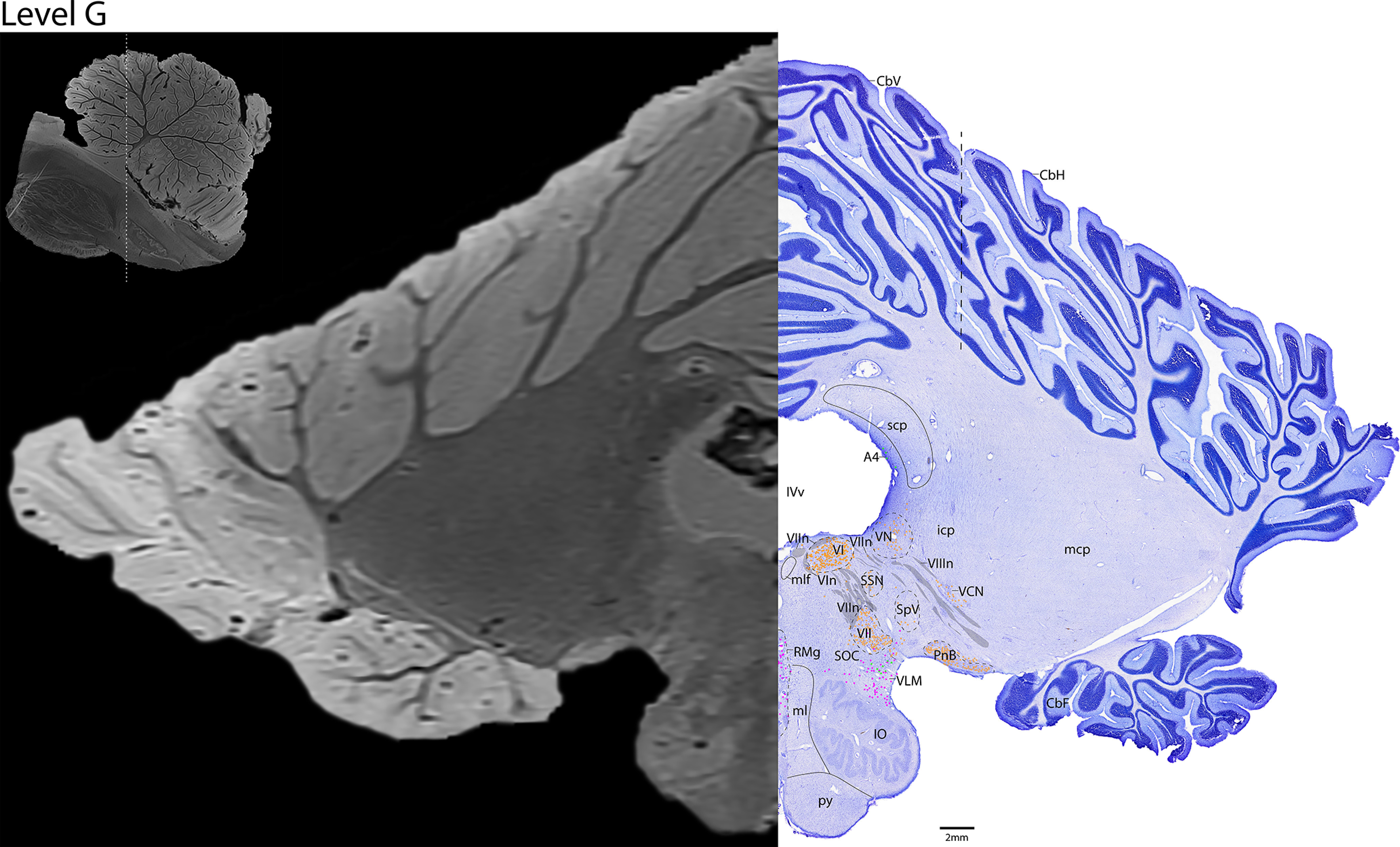
The ponto-medullary junction. Level G is comprised of MRI image on left and Nissl stain on right. Dots represent different cell types (orange represents ChAT; pink represents TpOH; bright green represents TH; dark green represents melanin-pigmented). Dashed line through sagittal section indicates the rostrocaudal level.

**Figure 19.**
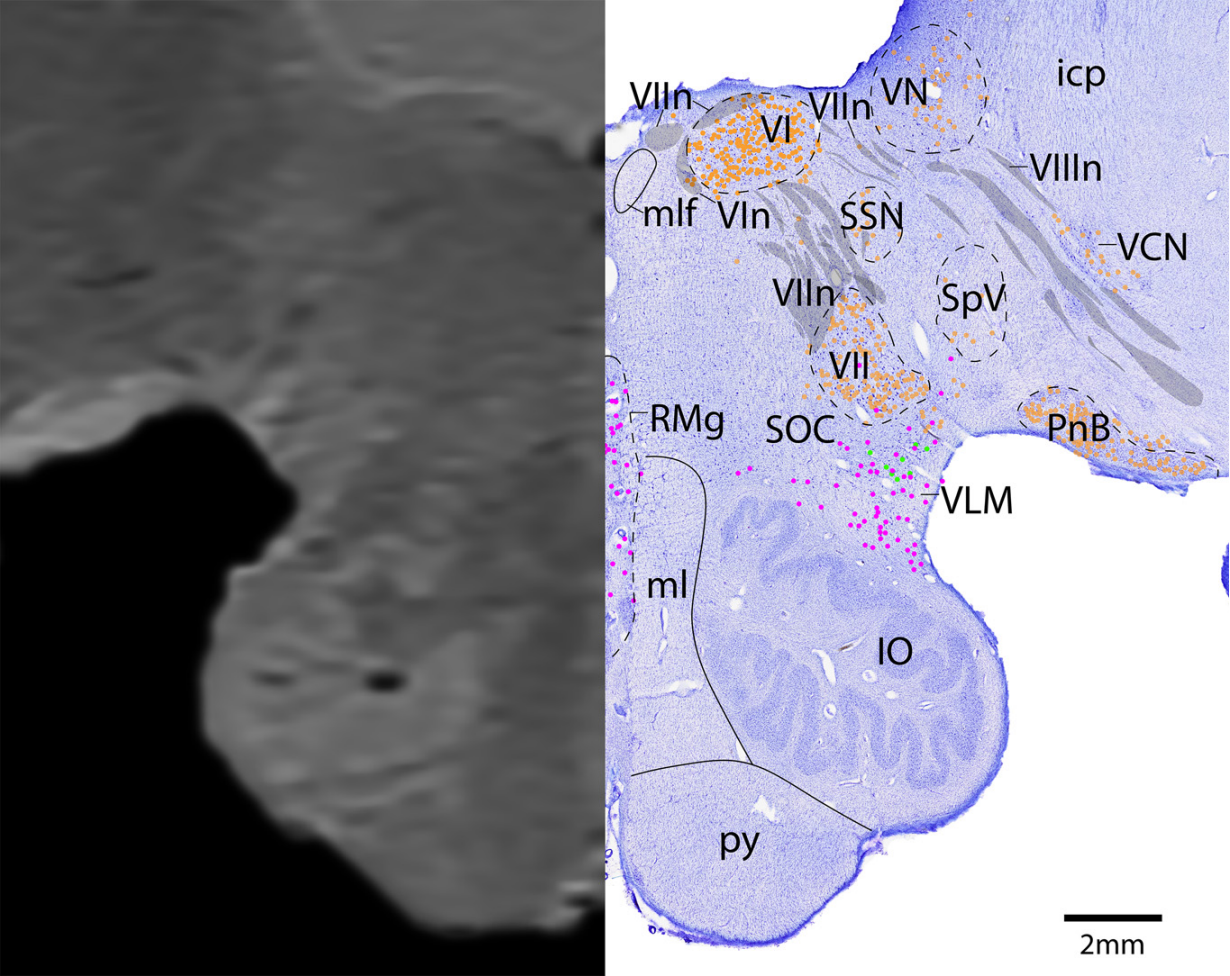
The ponto-medullary junction, a zoomed-in view. This is a zoomed-in view of the brainstem of Level G from Figure 18.

*Level G*: The ponto-medullary junction level (Figs. 18, 19) contains the large cholinergic motor neurons of cranial nuclei 6 and 7, the abducens nucleus (VI), and the facial nucleus (VII). The cholinergic fibers of the facial nerve (VIIn) can be seen exiting the facial nucleus dorsally and wrapping around VI. This level also contains the parasympathetic cholinergic neurons of the SSN, and dorsally, scattered cholinergic cells were found in the vestibular nuclei with the cholinergic fibers of the vestibulocochlear nerve (VIIIn). Lateral and ventral to VIIIn are the cholinergic neurons of the ventral cochlear nucleus (VCN) and pontobulbar nucleus, respectively. Serotonergic neurons are only located in the ventral portion of the brainstem at this level, in the raphe magnus (RMg) medially and ventrolateral medulla (VLM) laterally. This level also contains the largest portion of the IO, with the medial lemniscus (ml) and pyramidal tract (py) just medial. The cerebellar flocculus is located on the ventral aspect of the cerebellum.

**Figure 20.**
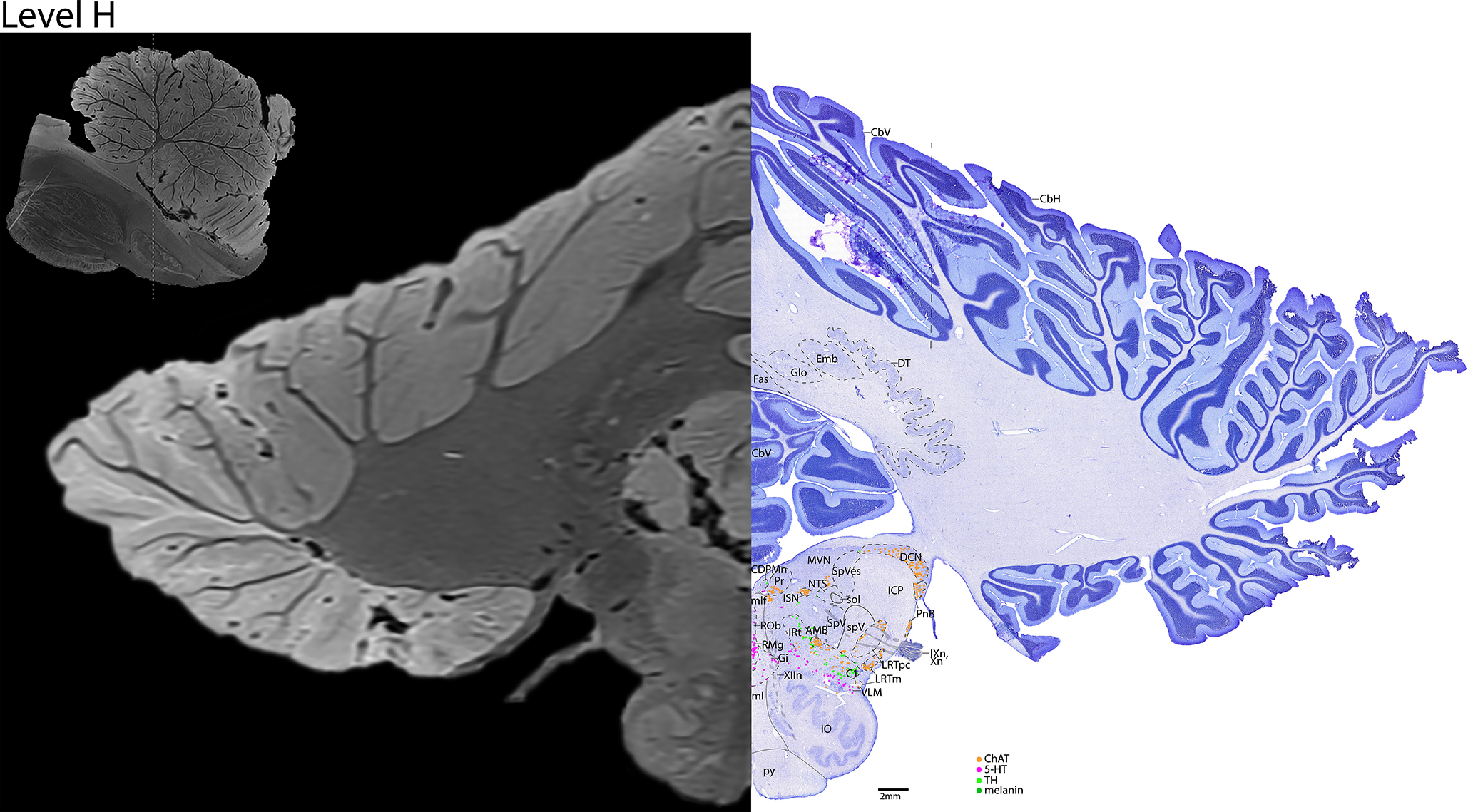
The rostral medulla. Level H is comprised of MRI image on left and Nissl stain on right. Dots represent different cell types (orange represents ChAT; pink represents TpOH; bright green represents TH; dark green represents melanin-pigmented). Dashed line through sagittal section indicates the rostrocaudal level.

**Figure 21.**
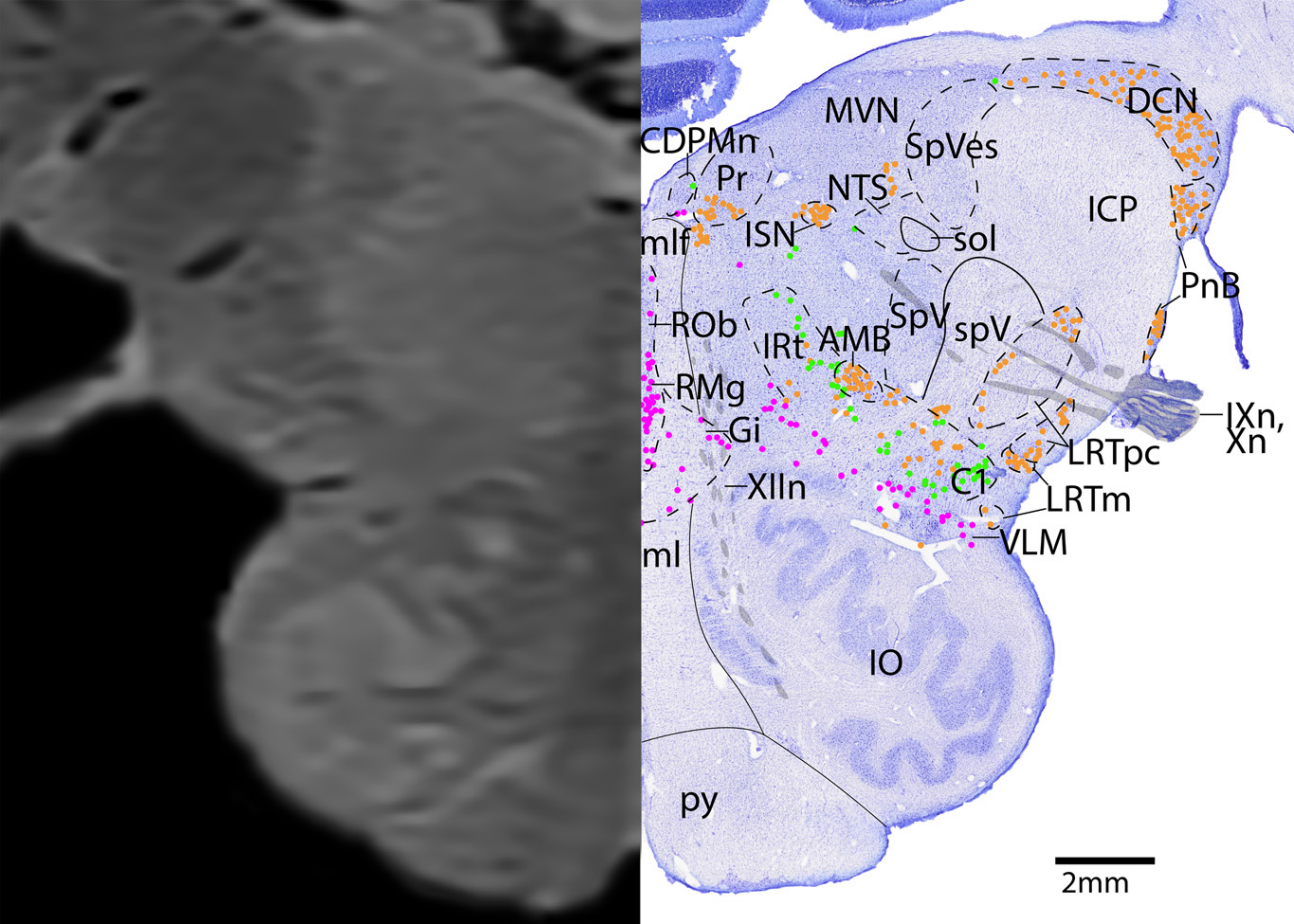
The rostral medulla, a zoomed-in view. This is a zoomed-in view of the brainstem of Level H from Figure 20.

*Level H*: The rostral medulla level (Figs. 20, 21) contains the cholinergic cranial nerves 9 and 10 (IXn, Xn) exiting the brain laterally, and cranial nerve 12 (XIIn) exiting ventromedially. Additional cholinergic groups include the dorsal cochlear nucleus and PnB dorsolaterally, the prepositus nucleus dorsomedially, the nucleus ambiguus (AMB) located within the catecholaminergic intermediate reticular zone (IRt), and the lateral reticular neurons (LRT) just dorsolateral to the IRt. The rostral NTS and solitary tract (sol) are at this level, with the cholinergic inferior salivatory nucleus (ISN) just medial to them. Additionally, the caudal midline serotonergic nuclei, including RMg and raphe obscurus (ROb), are present at this level, with the serotonergic neurons of the VLM extending laterally. The ventral brainstem at this level contains the IO laterally, and the ml and py medially. The cerebellar nuclei are prominent at this level (fastigial, globose, emboliform, dentate nuclei).

**Figure 22.**
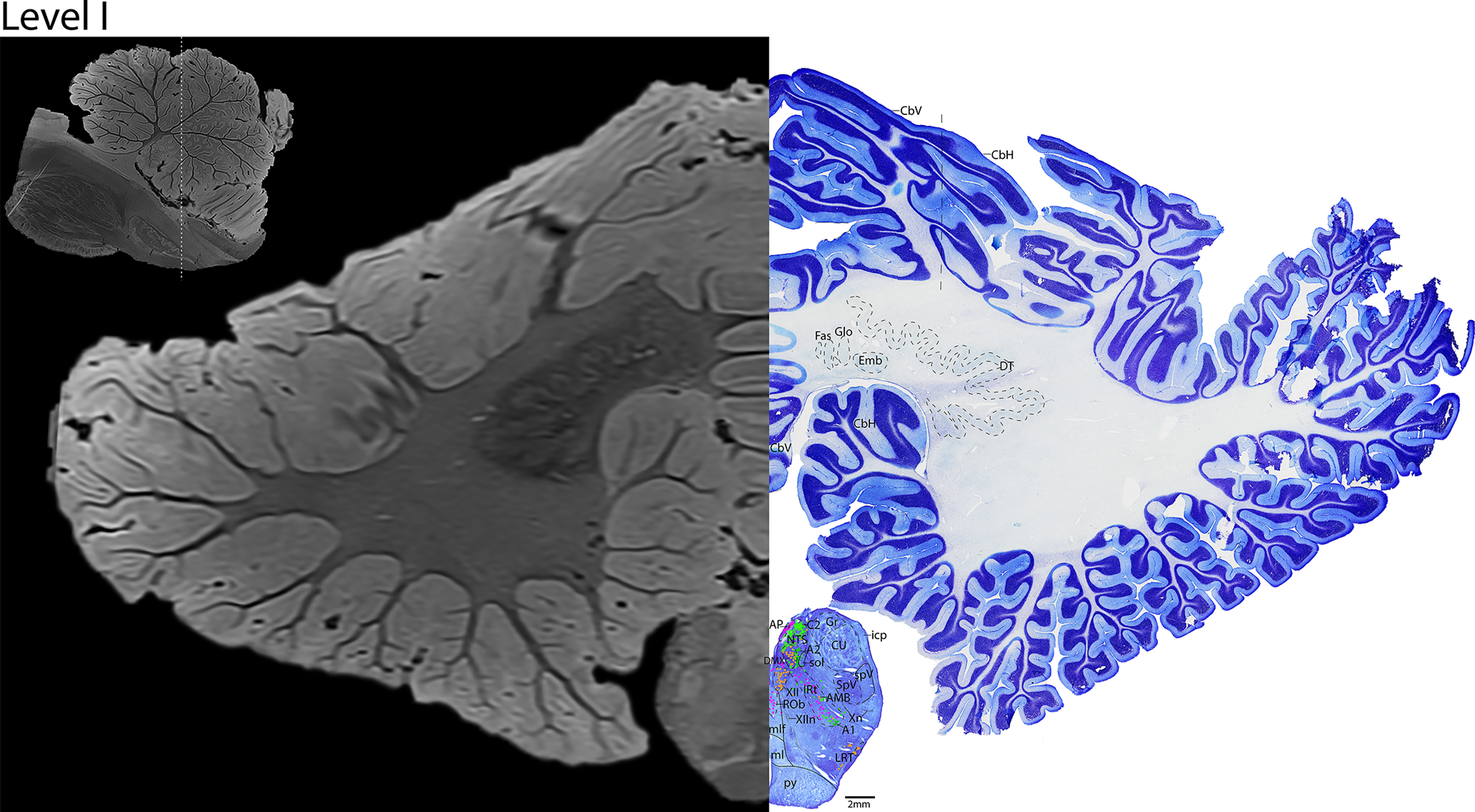
The mid-medulla. Level I is comprised of MRI image on left and Nissl stain on right. Dots represent different cell types (orange represents ChAT; pink represents TpOH; bright green represents TH; dark green represents melanin-pigmented). Dashed line through sagittal section indicates the rostrocaudal level.

**Figure 23.**
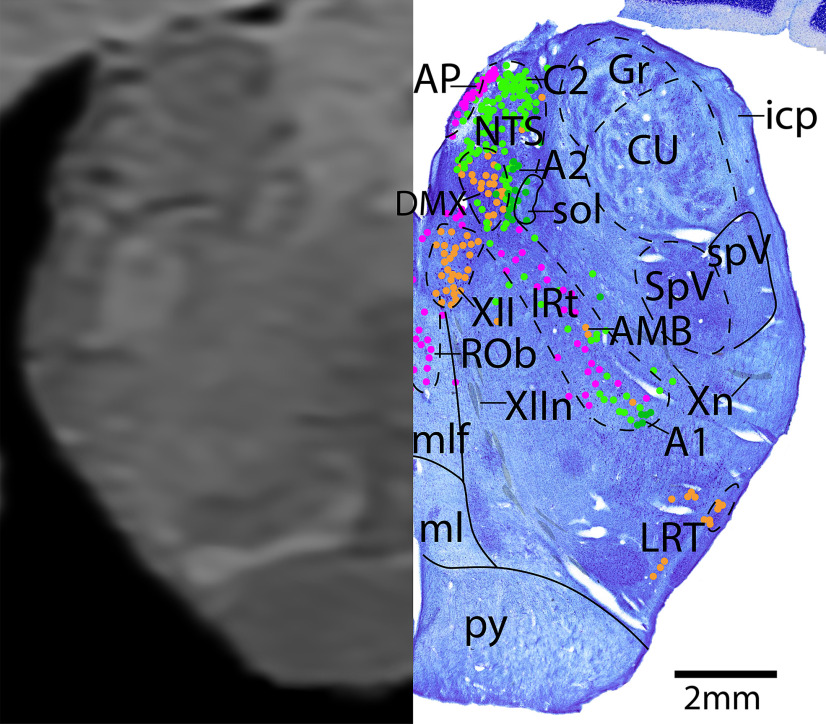
The mid-medulla, a zoomed-in view. This is a zoomed-in view of the brainstem of Level I from Figure 22.

*Level I*: The mid-medulla level (Figs. 22, 23) contains the densely packed adrenergic neurons of the NTS and C2, with the serotonergic (and to a lesser extent, adrenergic) area postrema just medial. Along the midline of the brainstem are the serotonergic neurons of the ROb. Extending diagonally is the IRt, and at this level it contains a mixture of serotonergic and adrenergic neurons, with few cholinergic AMB neurons. The medial cholinergic neurons are the motor neurons of the dorsal motor nucleus of vagus (DMX) and hypoglossal nucleus (XII), with the vagus nerve (Xn) extending laterally and XIIn exiting ventrally. The lateral cholinergic neurons are the LRT. Dorsolaterally are the spinal trigeminal nucleus (SpV) and spinal trigeminal tract (spV).

**Figure 24.**
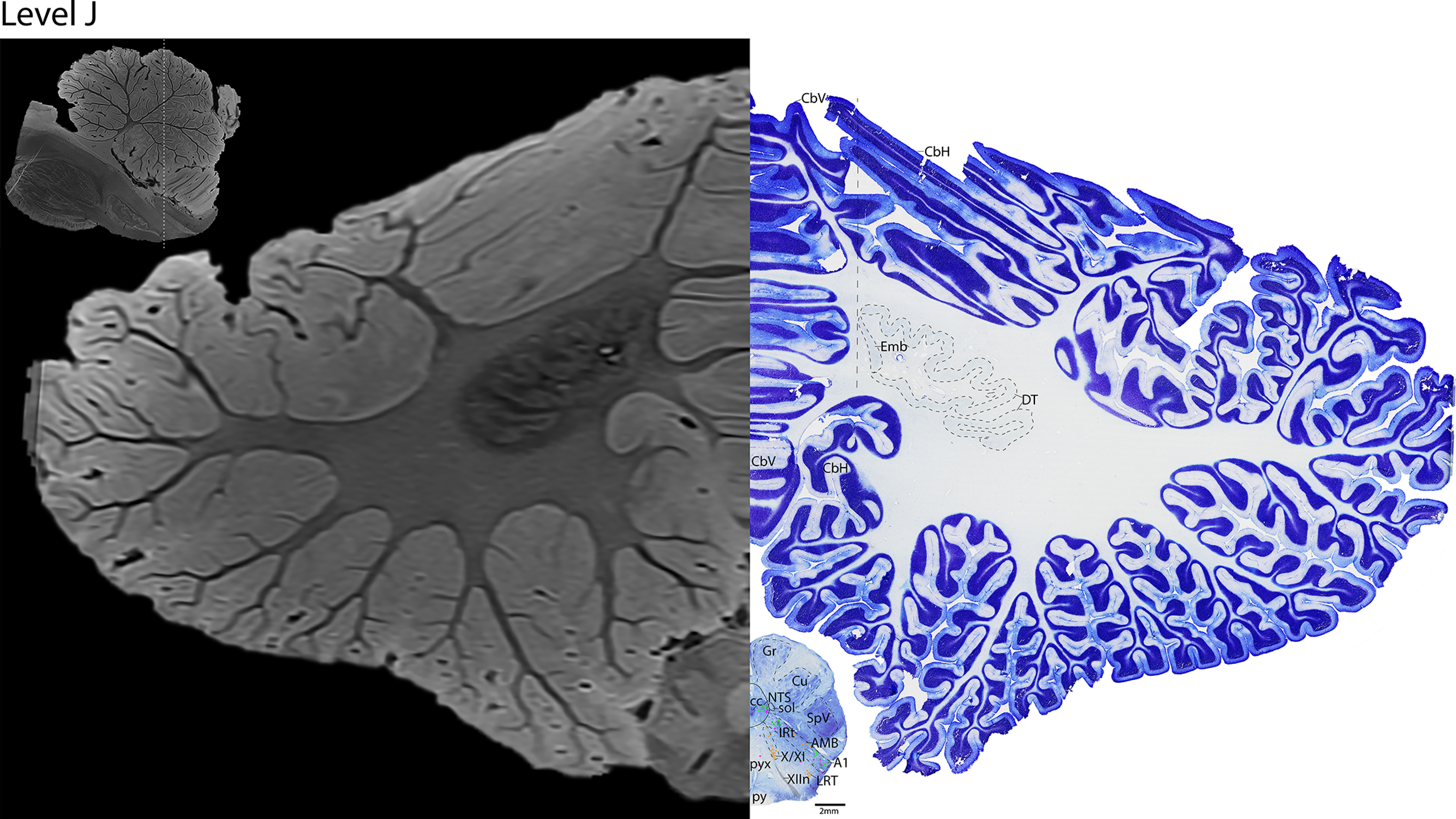
The caudal medulla. Level J is comprised of MRI image on left and Nissl stain on right. Dots represent different cell types (orange represents ChAT; pink represents TpOH; bright green represents TH; dark green represents melanin-pigmented). Dashed line through sagittal section indicates the rostrocaudal level.

**Figure 25.**
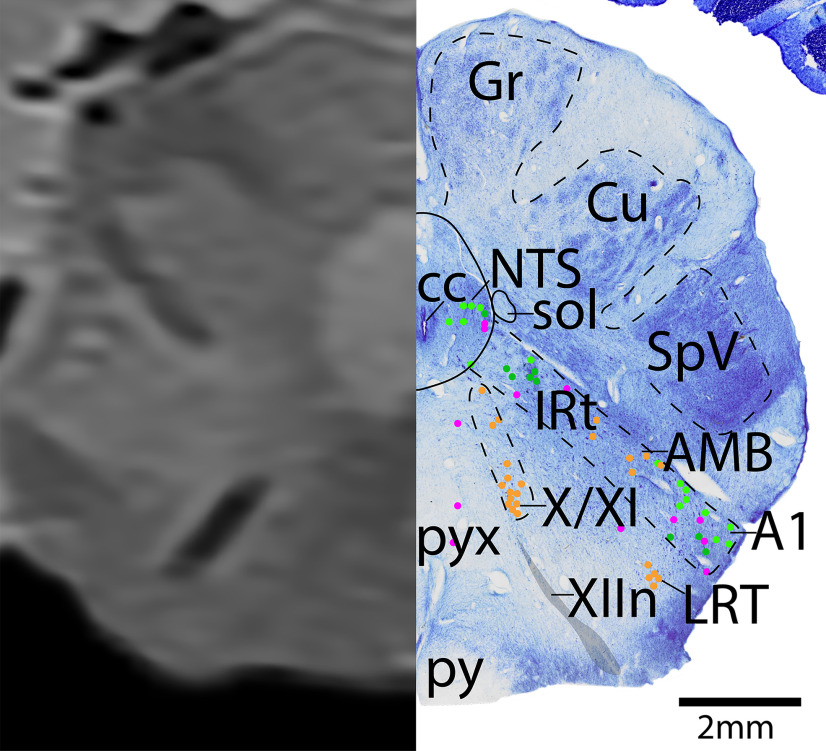
The caudal medulla, a zoomed-in view. This is a zoomed-in view of the brainstem of Level J from Figure 24.

*Level J*: The caudal medulla (Figs. 24, 25) contains the decussation of the pyramids (pyx). The caudal portion of the IRt and AMB are at this level, extending laterally to the adrenergic neurons of A1. The cholinergic vagal motor neurons and possible spinal accessory neurons (X/XI) can be seen just dorsal to the cholinergic XIIn that exits the ventral surface of the brain. Laterally, this level also has a more dense SpV and dorsal column nuclei (nucleus gracilis [Gr], and nucleus cuneatus [Cu]).

## Discussion

Clinicians are familiar with cross-sectional anatomy of the human brainstem from MRI scanners, but generally have had to rely on atlases which (1) lack a detailed view of brainstem cell groups as they appear in a Nissl stain, (2) lack the cerebellum for spatial reference, or (3) were not cut in the same plane (Sabin, 1901; Paxinos, 1995; Buttner-Ennever and Horn, 2014). We chose to cut the brainstem in the axial plane of section (and map these axial sections along the rostrocaudal extent of a sagittal view, Fig. 5) because the axial plane is most frequently used to view clinical MRI scans of human brainstems. The goal of this current atlas is to map the distribution of key brainstem cell types (cholinergic, serotonergic, and catecholaminergic neurons) in relationship to each other and to the main cell groups within the human brainstem and cerebellum, and also to pair this histology with 7T MR images. This project allows correlation of the chemoarchitecture with corresponding MRI of the brainstem, and makes the identification of cell groups that are often discussed, but rarely identifiable on MRI scan, accessible to clinicians and clinical researchers.

### Cholinergic groups

We identified robustly labeled ChAT^+^ cholinergic neurons in motor nuclei, preganglionic parasympathetic nuclei (preganglionic EW nucleus, salivatory nuclei), PPT, LDT, lateral parabrachial nucleus, and reticular formation. We found these cholinergic neurons in a similar distribution to previous reports in rat (Armstrong et al., 1983), cat (Kimura et al., 1981; Jones and Beaudet, 1987), and human brainstems (Mesulam et al., 1989), but our staining appears more extensive and the differences are described below.

#### Cranial nuclei and parasympathetics

The cranial nerve motor nuclei were identified by large, densely grouped ChAT^+^ motor neurons. There is not a clear separation between the cholinergic neurons of cranial nuclei III and IV, but the direction of the cholinergic fibers exiting from the cell bodies is informative. The oculomotor nucleus (III) was identified by large cholinergic somata in the midbrain periaqueductal region with ChAT^+^ fibers exiting ventrally (IIIn), as they eventually exit out of the base of the midbrain (Figs. 6, 7). The parasympathetic neurons of III, the preganglionic EW nucleus, were identified as the smaller ChAT^+^ neurons situated just dorsal to the more tightly clustered III. The trochlear nerve (IVn) was identified just caudally, as it coursed dorsally along the medial longitudinal fasciculus (mlf), and around the periaqueductal gray, and eventually above the fourth ventricle (IVv) where it decussated caudal to the midbrain (Figs. 8–11).

The motor trigeminal nucleus (V) was identified by the large cluster of cholinergic somata in the lateral pons (Figs. 16, 17) that gave rise to dense ChAT^+^ fiber bundles exiting ventrolaterally through the middle cerebellar peduncle (mcp) (Figs. 12–17). The principle sensory trigeminal nucleus was identified by the patchy ChAT^+^ neuropil just lateral to V and just rostral to the spinal trigeminal nucleus (SpV).

The abducens nucleus (VI) and the facial nucleus (VII) contain large, ChAT^+^ motor neurons, and can be seen on the same level, Level G (Figs. 3, 18, 19). The abducens nucleus is comprised of both ChAT^+^ cholinergic neurons and neurons that are not ChAT^+^ (Fig. 3). The latter are presumably the abducens internuclear neurons that project contralaterally through the ChAT^–^ medial longitudinal fasciculus (mlf) to the opposite III motor neurons and medial rectus to yoke the eyes in horizontal movement. The axons of the facial nerve are also robustly ChAT^+^ and can be seen exiting the facial nucleus dorsally, approaching the abducens nucleus ventromedially and wrapping over and around it, and traveling ventrolaterally before exiting the brain (Figs. 3, 18, 19).

Between the motor trigeminal nucleus (V) and facial nucleus (VII) is a continuum of neurons that comprise the accessory trigeminal and accessory facial neurons (acc V/VII, Figs. 16, 17). In rodents, the rostral part of this accessory continuum (acc V) innervates the tensor tympani, mylohyoid, and anterior belly of the digastric, while the caudal portion (acc VII) innervates the stapedius, stylohyoid, and posterior belly of the digastric (Szekely and Matesz, 1982; Shohara and Sakai, 1983). The neurons in the accessory nuclei are large like the primary trigeminal and facial motor neurons, and likely extend caudally as the unlabeled ChAT^+^ neurons within the facial nerve between the facial and abducens nuclei (Figs. 18, 19).

We delineated the salivatory nuclei based on ChAT^+^ staining and the spatial relationship to other known nuclei based on previous tract tracing studies (Brown and Howlett, 1968; Hiura, 1977; Satomi et al., 1979a; Contreras et al., 1980; Spencer et al., 1990). The SSN is comprised of preganglionic cholinergic neurons that project via the facial nerve (some via the greater petrosal nerve) to the pterygopalatine ganglion, which innervates submandibular, sublingual, and lacrimal glands, and cerebral blood vessels (Contreras et al., 1980; Spencer et al., 1990; Nakai et al., 1993). The SSN has been located in rodents and cats by retrogradely labeling neurons from the pterygopalatine ganglion and the facial nerve (Brown and Howlett, 1968; Hiura, 1977; Satomi et al., 1979a; Contreras et al., 1980; Spencer et al., 1990). They consist of cholinergic neurons dorsolateral to the facial nucleus along the ascending facial nerve (Figs. 18, 19). These parasympathetic SSN cells have smaller somata than the motor neurons of the facial nucleus proper.

The ISN (Figs. 20, 21) contains cholinergic preganglionic neurons that project via the glossopharyngeal nerve (IXn) (lesser petrosal nerve) to the otic ganglion, which in turn innervates the parotid gland. The location of the inferior salivatory neurons have been worked out in rat and cat via retrograde tracing from the glossopharyngeal and vagus nerves, and appear to intermingle with the retrogradely labeled neurons from the facial nerve branches that define the SSN (Satomi et al., 1979a,b; Contreras et al., 1980). At least in the rat, the ISN is adjacent to the lingual-tonsillar branch of IXn caudally at the level of the rostral NTS (Contreras et al., 1980); we labeled the cholinergic neurons in this area as ISN in the human brainstem section at this level (Figs. 20, 21). Furthermore, in human studies, the ISN was labeled as AChE^+^ cells at the rostral tip of the DMX, and dorsomedial to the NTS (Braak, 1972; McRitchie and Tork, 1993); we found ChAT^+^ neurons in a similar location just medial to the rostral NTS. These robustly stained ChAT^+^ neurons are tightly clustered with a dense ChAT^+^ neuropil, and have very prominent TpOH fiber staining specifically limited to the region of cholinergic neurons. By contrast, TpOH is not present over the colinergic neurons rostrally in the SSN (Figs. 18, 19).

Lateral to the ISN is the NTS and the solitary tract (sol) (Figs. 20, 21). At this level, the NTS is marked by a ChAT^+^ neuropil indicating the medial subdivision of the NTS. For more details about the NTS, see Catecholaminergic (dopaminergic and adrenergic) groups.

The AMB contains cholinergic motor neurons that project to the larynx and pharynx, and also contains smaller preganglionic parasympathetic neurons which decrease heart rate via cranial nerves 9 and 10. We identified AMB neurons within the medullary reticular formation as the more compact ChAT^+^ neurons within a ChAT^+^ neuropil (Figs. 20, 21). These neurons are larger, more robustly labeled, and more tightly clustered than the other surrounding cholinergic neurons. We hypothesize that these AMB neurons likely represent the compact part of the AMB, which according to a detailed study in rat, mainly contains cells innervating the esophagus (Bieger and Hopkins, 1987). AMB extends caudally, although it is less densely clustered at these levels (Figs. 22–25).

In the medial medulla, the DMX and the hypoglossal nucleus (XII) can be seen at the same level (Figs. 4, 22, 23). The robustly ChAT^+^ fibers of cranial nerve 12 (XIIn) can be seen traveling ventrally, just lateral to the midline. The large, ChAT^+^ somata of DMX can be seen in Figure 4. Additionally, some of the unlabeled ChAT^+^ neurons marked at the ventral aspect of the prepositus nucleus (Figs. 20, 21) are likely neurons of the rostral hypoglossal nucleus.

We marked the spinal accessory nucleus (XI), the nucleus of cranial nerve 11, as the cholinergic neurons located lateral to the pyramids, and dorsal to the cholinergic fibers of XIIn exiting the brain (Figs. 24, 25). However, a previous study in monkeys found that the vagal motor neurons innervating the infrahyoid muscles (the sternohyoid and sternothyroid muscles, rostrally, and the omohyoid, caudally) form the rostral end of a continuum with the neurons that innervate the spinal accessory muscles (sternocleidomastoid and trapezius muscles via CN 11) (Ueyama et al., 1990). This continuum of cells begins in the medulla rostral to the decussation of the pyramids, and continues caudally into the cervical spinal cord. The medullary section at Level J (Figs. 24, 25) appears relatively rostral in the continuum of cells given that the pyramidal decussation and cholinergic neurons are adjacent to the hypoglossal nerve, suggesting that these rostral cholinergic neurons may be mostly vagal motor neurons. Without retrograde tracing studies we cannot be sure which of these cholinergic neurons innervate infrahyoid muscles or spinal accessory muscles, so we labeled them all as X/XI to indicate that they may include both vagal motor neurons (X) and spinal accessory neurons (XI).

#### PPT and LDT

We found a distribution of cholinergic neurons in the PPT and LDT, similar to that previously reported in human, cat, and rat brainstems (Armstrong et al., 1983; Jones and Beaudet, 1987; Mesulam et al., 1989; Manaye et al., 1999). The majority of cholinergic neurons in this region were localized to the more lateral PPT (Figs. 8, 9, with the sparse yet caudalmost portion of the PPT on Figs. 10, 11), and fewer comprised the LDT in the central gray matter (Figs. 10, 11). Dorsal LDT neurons intermingle with adrenergic LC neurons (Figs. 10, 11).

#### Parabrachial nucleus

The parabrachial nuclei are caudal to the decussation of the scp, which appears as a crescent in the dorsolateral pons, lateral to the fourth ventricle (IVv). At this level of the caudal scp, we labeled the lateral (PBl) and medial parabrachial nuclei as the neurons that are lateral and medial to the caudal scp, respectively (Figs. 10–17). The ventrolateral portions of the PBl contained cholinergic neurons caudally (Figs. 14, 15), and we labeled this the external lateral parabrachial nucleus. This region also contains CGRP^+^ neurons (de Lacalle and Saper, 2000), and we know from rat studies that the CGRP^+^ neurons are adjacent to (but separate from) the ChAT^+^ neurons in the external lateral parabrachial nucleus.

#### Reticular formation

Within the reticular formation, we labeled the swath of intermingled cholinergic, serotonergic, and catecholaminergic neurons as the IRt (Figs. 20–25). We found a similar distribution of TH^+^ labeling to what had previously been described in this region (Pearson et al., 1983; Arango et al., 1988; Halliday et al., 1988). Along the IRt, we found darkly labeled TH^+^ neurons intermixed with lightly labeled ChAT^+^ neurons, and their relationship to serotonergic neurons varies along the rostrocaudal extent. Rostrally in the IRt, we found that cholinergic and TH^+^ neurons were just dorsal and adjacent to the separate and robustly labeled serotonergic neurons of the VLM (Figs. 20, 21). This distinct distribution is different from the arrangement of overlapping adrenergic and serotonergic neurons presented by Halliday et al. (1988) who used phenylalanine hydroxylase^+^ (PH8) to label serotonergic neurons. However, we found that these separate populations merged more caudally in the IRt/VLM where serotonergic neurons intermixed with TH^+^ and ChAT^+^ neurons (Figs. 22, 23).

Within the IRt, we designated the more compact distribution of ChAT^+^ neurons within a ChAT^+^ neuropil as nucleus AMB (Figs. 20, 21). These neurons are larger, more robustly labeled, and more tightly clustered than the other surrounding cholinergic neurons. We hypothesize that these AMB neurons likely represent the compact part of the AMB, which according to a detailed study in rat, may contain cells innervating the esophagus (Bieger and Hopkins, 1987).

#### Precerebellar nuclei

The LRT are cholinergic precerebellar neurons, and are difficult to pinpoint in a human brain because they are defined by their efferent projections to the cerebellum. Since we do not have tracing data in our human brains, we estimated the location of these neurons using cholinergic staining, cytology, and landmarks. We marked the LRT neurons as the small, tight clusters of generally larger, robustly ChAT^+^ neurons that avoided C1, and are above and below the IRt and VLM (Figs. 17–22). Previously, Walberg (1952) detailed the cytoarchitecture of the LRT across numerous mammals, and he generally used the nomenclature “main portion” (pars principalis) and “subtrigeminal portion.” Within the main portion, he noted that many species have a magnocellular (LRTm) and parvicellular (LRTpc) part where the cells are predominantly larger or smaller, respectively. Adapting Walberg's nomenclature, we labeled the oval/elliptical neuropil with occasional small ChAT^+^ somata as “LRTpc” in the lateral pons (Figs. 20, 21). Ventral to the LRTpc, there are much larger and more darkly stained ChAT^+^ neurons that we have labeled as magnocellular, LRTm.

The pontobulbar nucleus was identified on the lateral edge of the medulla by its dense ChAT^+^ neuropil that obscures the view of the small ChAT^+^ somata (Figs. 18–21). The PnB was distinguished from the VCN, which had weaker ChAT^+^ somata and faint ChAT^+^ neuropil (Figs. 18, 19). In contrast to the VCN, the dorsal cochlear nucleus superiorly had more robust ChAT^+^ somata and neuropil (Figs. 20, 21). A similar pattern has been described in cats (Godfrey et al., 1977), but the VCN the of rat might have more robust cholinergic staining than its human or cat counterparts (Godfrey and Matschinsky, 1981).

### Catecholaminergic (dopaminergic and adrenergic) groups

Catecholaminergic neurons (dopaminergic and adrenergic) have been described in the brains of many species. We used the A1-A14 nomenclature from Dahlstrom and Fuxe (1964) especially in cases where there is not a more widely accepted name (e.g., the large A4 neurons found in the lateral wall and roof of the fourth ventricle) (Figs. 16–18).

Catecholaminergic neurons can be identified by both the presence of the endogenous pigmented melanin and immunohistochemical staining for the enzyme TH. Prior reports have estimated that only 50%–65% of medullary catecholaminergic neurons produce pigment, implying that quantifying only pigmented neurons would underestimate the number of catecholaminergic cells (Halliday et al., 1988; Huang et al., 1993). Additionally, we found many melanin-pigmented neurons that are TH^–^, suggesting that analyzing TH^+^ neurons alone would also underestimate the number of catecholaminergic neurons. Therefore, we decided to use both TH staining and endogenous pigmented melanin to detect the maximal number of catecholaminergic neurons in our study.

We are currently unaware of functional differences between neurons that are marked by either TH or melanin alone, or those that contained both. Given that melanin is a byproduct of catecholamine synthesis (Graham, 1979), one would expect to see melanin in TH^+^ neurons. We are currently unsure why some neurons are only either TH^+^ or melanin-pigmented (but TH^–^); perhaps they no longer produce high enough levels of the TH enzyme for IHC detection (as a neuron fills with melanin, its synthetic ability for making more catecholamines might become impaired), or they do not yet contain a visibly detectable amount of melanin since adult brains seem to develop melanin in these regions over a lifetime. More research is required in this area.

However, we did notice patterns between TH and melanin pigmentation in both staining quality and spatial distribution. In general, we found that the TH^+^ neurons that are nonpigmented stain darker for TH than the TH^+^ cells that contain pigmented melanin, and the darkest pigmented melanin neurons appear to be TH^–^ (or their TH levels are too low to detect).

Overall, some brainstem catecholaminergic neurons contained both pigment and TH, such as in the adrenergic LC, as only a few pigmented cells in the LC seemed to be TH^–^. This is demonstrated in Figure 2*D*, where the brownish melanin is surrounded by blackish gray TH^+^ stain, and in Figures 10–17 where there are mostly lime green dots representing TH^+^ stain, rather than dark green representing melanin pigment alone. In the A8 group (Figs. 6, 7), most of the cells were both pigmented and TH^+^, but especially dorsally, there were some neurons that stained darker with TH that were nonpigmented.

Additionally, there are a few nuclei with cells that are either predominately TH^+^ or pigmented, but the two labels are not colocalized. For example, the lateral and ventral parts of the SN contains mostly robustly pigmented neurons that are not labeled by TH (Figs. 6–9), which has also been reported by an earlier study (Pearson et al., 1983). Additionally, all of the TH^+^ cells superior to A8 are robustly labeled with TH (Figs. 6, 7) but are without pigment. Perhaps this pattern is related to the different catecholaminergic cell types, given the SN is dopaminergic, while neurons dorsal to A8 are likely adrenergic.

We found clusters of TH^+^ neurons in the ventrolateral portion of the IRt, which we have labeled C1 rostrally (Figs. 17, 18) and A1 caudally (Figs. 19–22), as previously described (Saper and Petito, 1982; Arango et al., 1988). In the IRt and C1 of the rostral medulla, the TH^+^ neurons do not appear to be pigmented (Figs. 20, 21). However, more caudally where we labeled A1, the TH^+^ neurons of the IRt are pigmented, although there also are pigmented neurons that are TH^–^ (Figs. 22–25). For further discussion of TH^+^ neurons in the IRt, see Cholinergic groups.

In the medial medulla around the DMX, there is a mixed population of catecholaminergic neurons (Figs. 4, 22, 23). Dorsally, in the NTS/C2, the neurons are medium-sized, robustly TH^+^, and either weakly pigmented or nonpigmented, with a few robustly pigmented neurons on the lateral border (Fig. 4*C*). More ventrally, in the dorsal and medial aspects of the DMX, there are smaller, more weakly TH^+^ neurons that are also either weakly pigmented or nonpigmented (Fig. 23). Lateral to the DMX and NTS, there are robustly pigmented TH^–^ neurons that we labeled as A2. This finding of TH^+^ neurons (A2) dorsal and medial to DMX and smaller, robustly melanin-pigmented neurons lateral to DMX is consistent with prior work (Saper and Petito, 1982; Huang et al., 1993).

### Serotonergic groups

An earlier study using Nissl stain on human brainstem parsed out subdivisions of the DR nucleus (Baker et al., 1991). Based on cell morphology, this study identified four DR subgroups. While we also observed some of their findings (e.g., that fusiform-shaped cells are more often found in the midline of the DR), we found that, after staining for TpOH, these four subgroups were less obviously delineated. We also did not observe their finding that lipofuscin pigmented cells were limited to the dorsal DR. Indeed, we found the ventral portion of the DR to be distinguished by slightly smaller cells with weaker TpOH staining but darker lipofuscin pigment and a slightly denser neuropil. Thus, for simplicity, we subdivided the large middle level into DRd and DRv where we saw this distinction (Figs. 8, 9), but we labeled the rostral and caudal most portions of the DR with just “DR,” given less obvious differences at these levels (Figs. 6, 7, 10, 11). Additionally, we found the lipofuscin pigment to be almost exclusively in the serotonergic DR cells as opposed to the nonserotonergic raphe cells.

Outside of the DR, we tried to keep the nomenclature of pontine serotonergic groups similar to previous work. Consistent with prior reports, we labeled the smaller midline neurons as MnR (Figs. 6–17); and laterally, we labeled the larger, ventral neurons along the lateral lemniscus as SuL (Figs. 10–15) (Dahlstrom and Fuxe, 1964; Baker et al., 1991; Hornung, 2003). Lateral to the MnR, there are more diffusely scattered serotonergic neurons that have been called nucleus pontis oralis or nucleus reticularis pontis oralis; but because of the heterogeneous and diffuse nature of this region, we referred to them as the PRF (Figs. 10–17).

We found the caudal midline serotonergic nuclei, including RMg, raphe pallidus, and ROb, to be a continuum of neurons with indistinct borders, rather than discrete nuclei (Figs. 8–23). However, to preserve prior nomenclature, we labeled the large portion of midline neurons at the level of the rostral and middle IO as RMg, and the more caudal midline groups dorsal to the medial lemniscus as ROb (dorsally) and RMg (ventrally), and RPa ventral to the lemniscus (Halliday et al., 1988; Hornung, 2003).

The VLM contains a robustly stained group of large serotonergic neurons mixed with a few TH^+^ neurons and sparse cholinergic neurons (Figs. 18–21). These serotonergic VLM neurons are also referred to as the lateral paragigantocellular reticular nucleus in some texts (Hornung, 2003; Buttner-Ennever and Horn, 2014).

### Conclusion

Our atlas provides a unique resource in coregistering a high-resolution MRI scan of the human brainstem and cerebellum with immunohistochemistry for three key neurotransmitter systems, creating an atlas of the cell groups of the brainstem. This set of data will allow future investigators and clinicians to register their MRI scans to this atlas, and improve segmentation of specific cell groups in MR studies.

#### Limitations

Although this study has novel features, there are also limitations worth discussing. As the work was completed in cadaveric tissue, air artifact became a variable to address with MR imaging, particularly with the use of SWI sequences. Although SWI provides some of the highest resolution in distinguishing gray-white matter differentiation at high resolution, it is particularly affected by air artifact. As such, we used agar as a medium to submerge the tissue into and occupy the fourth ventricle and cerebral aqueduct to displace air inside and around the tissue. While the agar largely minimized air artifact, it did introduce some distortion of tissue, particularly of the obex and central canal more caudally. This presented some inaccuracy in the coregistration between the histology and MRI. Future work to adapt nonlinear registration techniques to population-based neuroanatomy may provide means to correct these distortions.

Next, this brainstem and cerebellum atlas was derived from a single specimen, which provides both benefits and limitations. We find our single subject to be a relative strength because all the sections are in register, the cells marked are of exact neurons and not an estimation; and consistent with the practice of most classic atlases, the data come from a single case of continuous, serial sections. However, given that the brainstem is one specimen, the histology could only be cut in one plane of section (while the MRI can be virtually cut in any and multiple planes). We chose the axial plane of section for histology given this is the most frequently viewed plane clinically. Additionally, new MRI studies are using large datasets, which was out of the scope of our study given we did not want the MRI to be an estimation but rather a representation of our exact histology. We also picked a case without known brainstem pathology, and compared the brainstem with others in our laboratory to pick a representative case.

Additionally, in some cases, the brainstem was satisfactorily connected to the cerebellum via the peduncles on the 40 μm section; in others (particularly rostral pons and caudal medulla), the brainstem is not structurally connected to the cerebellum. When mounting the tissue on the slide, every effort was used to maintain the anatomic spatial relationship between the brainstem and cerebellum that was not structurally connected; however, in some cases, this was not identically executed. This therefore introduced some error in the MRI image exactly aligning with the histologic counterpart.

In conclusion, this atlas is unfortunately missing the anterior portion of the midbrain as a consequence of the autopsy protocol, which severed the fresh brainstem from the forebrain by a knife cut caudal to the mammillary bodies. We included the most rostral portion of the midbrain that we had as Level A. However, the dorsal part of the brainstem was not available at that level.

## Appendix

A1-A14, catecholaminergic groups (nomenclature from nomenclature from Dahlstrom and Fuxe 1964) acc V/VII, accessory trigeminal and accessory facial neurons

AMB, nucleus ambiguus

AP, area postrema

Aq, cerebral aqueduct

C1, catecholaminergic group

C2, catecholaminergic group

CbF, cerebellar flocculus

CbH, cerebellar hemisphere

CbV, cerebellar vermis

CbVg, cerebellar vermis granular cell layer

CbVm, cerebellar vermis molecular layer

CbVp, cerebellar vermis Purkinje cell layer

CDPMn, caudal dorsal paramedian nucleus

CGRP, calcitonin gene-related peptide

ChAT, choline acetyl transferase

Cu, nucleus cuneatus

DCN, dorsal cochlear nucleus

DMX, dorsal motor nucleus of vagus

DR, dorsal raphe

DRd, dorsal subdivision of dorsal raphe

DRv, ventral subdivision of dorsal raphe

DT, dentate nucleus

DTg, dorsal tegmental nucleus

Emb, emboliform nucleus

EW, Edinger-Westphal nucleus

Fas, fastigial nucleus

Glo, globose nucleus

Gr, nucleus gracilis

ICol, inferior colliculus

icp, inferior cerebellar peduncle

III, oculomotor nucleus

IIIn, oculomotor nerve/ third cranial nerve

IO, inferior olive

IPN, interpeduncular nucleus

IRt, intermediate reticular zone

ISN, inferior salivatory nucleus

IV, trochlear nucleus

IVn, trochlear nerve/ fourth cranial nerve

IVv, fourth ventricle

IX, glossopharyngeal nerve nucleus

IXn, glossopharyngeal nerve, ninth cranial nerve

LC, locus coeruleus

LDT, laterodorsal tegmentum

LL, lateral lemniscus

LPGi, lateral paragigantocellular reticular nucleus

LRT, lateral reticular neurons

LRTm, lateral reticular neurons magnocellular part

LRTpc, lateral reticular neurons parvicellular part

mcp, middle cerebellar peduncle

Me5, mesencephalic trigeminal nucleus

me5, mesencephalic trigeminal tract

ml, medial lemniscus

mlf, medial longitudinal fasciculus

MnR, midline median raphe

NTS, nucleus of the solitary tract

PAG, periaqueductal gray

PBel, external lateral parabrachial nucleus

PBG, parabigeminal nucleus

PBl, lateral parabrachial nucleus

PBm, medial parabrachial nucleus

PnB, pontobulbar nucleus

PnO, pontis oralis

PPT, pedunculopontine tegmentum

Pr, prepositus nucleus

PRF, pontine reticular formation

PSV, principle sensory trigeminal nucleus

py, pyramidal tract

pyx, pyramidal tract decussation

RMg, raphe magnus

ROb, raphe obscurus

SCol, superior colliculus

scp, superior cerebellar peduncle

SN, substantia nigra

sol, solitary tract

SOC, superior olivary complex

SpV, spinal trigeminal nucleus

spV, spinal trigeminal tract

SpVes, spinal vestibular nucleus

SSN, superior salivatory nucleus

SuL, supralemniscal/B9

TH, tyrosine hydroxylase

TpOH, tryptophan hydroxylase

V, trigeminal motor nucleus

VCN, ventral cochlear nucleus

Vn, trigeminal nerve/ fifth cranial nerve

VI, abducens nucleus

VII, facial nucleus

VIIIn, vestibulocochlear nerve

VIIn, facial nerve/ seventh cranial nerve

VIn, abducens nerve/sixth cranial nerve

VLM, ventrolateral medulla

Vn, trigeminal nerve/ fifth cranial nerve

VN, vestibular nuclei

VTA, ventral tegmental area

X, vagal motor neurons

XI, spinal accessory nucleus

XII, hypoglossal nucleu

XIIn, hypoglossal nerve, twelfth cranial nerve

XIn, XI, spinal accessory nerve, eleventh cranial nerve
